# Systematic review of early abortion services in low- and middle-income country primary care: potential for reverse innovation and application in the UK context

**DOI:** 10.1186/s12992-020-00613-z

**Published:** 2020-09-30

**Authors:** Jacy Zhou, Rebecca Blaylock, Matthew Harris

**Affiliations:** 1 School of Public Health, Imperial College London, London, UK; 2 British Pregnancy Advisory Service, London, UK

**Keywords:** Abortion, Quality of service, Mid-level providers, Low-and-middle-income countries, Reverse innovation, UK

## Abstract

**Background:**

In the UK, according to the 1967 Abortion Act, all abortions must be approved by two doctors, reported to the Department of Health and Social Care (DHSC), and be performed by doctors within licensed premises. Removing abortion from the criminal framework could permit new service delivery models. We explore service delivery models in primary care settings that can improve accessibility without negatively impacting the safety and efficiency of abortion services. Novel service delivery models are common in low-and-middle income countries (LMICs) due to resource constraints, and services are sometimes provided by trained, mid-level providers via “task-shifting”. The aim of this study is to explore the quality of early abortion services provided in primary care of LMICs and explore the potential benefits of extending their application to the UK context.

**Methods:**

We searched MEDLINE, EMBASE, Global Health, Maternity and Infant Care, CINAHL, and HMIC for studies published from September 1994 to February 2020, with search terms “nurses”, “midwives”, “general physicians”, “early medical/surgical abortion”. We included studies that examined the quality of abortion care in primary care settings of low-and-middle-income countries (LMICs), and excluded studies in countries where abortion is illegal, and those of services provided by independent NGOs. We conducted a thematic analysis and narrative synthesis to identify indicators of quality care at structural, process and outcome levels of the Donabedian model.

**Results:**

A total of 21 indicators under 8 subthemes were identified to examine the quality of service provision: law and policy, infrastructure, technical competency, information provision, client-provider interactions, ancillary services, complete abortions, client satisfaction. Our analysis suggests that structural, process and outcome indicators follow a mediation pathway of the Donabedian model. This review showed that providing early medical abortion in primary care services is safe and feasible and “task-shifting” to mid-level providers can effectively replace doctors in providing abortion.

**Conclusion:**

The way services are organised in LMICs, using a task-shifted and decentralised model, results in high quality services that should be considered for adoption in the UK. Collaboration with professional medical bodies and governmental departments is necessary to expand services from secondary to primary care.

## Introduction

In England and Wales, the criminalisation of abortion persists as a source of stigma, discrimination against women, and hinders provision of patient-centred clinical practices [1]. According to the 1967 Abortion Act, all abortions must be approved by two doctors, reported to the Department of Health and Social Care (DHSC), and can only be performed by doctors within licensed premises [2]. This legal framework causes accessibility issues, especially in rural communities lacking in both medical facilities and providers, and prevents the development of other innovative models, such as nurse/midwife-led surgical services [3]. The UK is unable to implement the WHO recommendation for services to optimise health worker roles in healthcare systems such as primary care because of the legal restrictions [4]. At the 1994 Cairo International Conference on Population and Development (ICPD), over 180 governments agreed that reproductive health care should be an integral part of primary health care and should be accessible in all countries “to all individuals of appropriate ages as soon as possible and no later than 2015” [5].

Several professional medical bodies, including the British Medical Association (BMA), the Royal College of Gynaecologists and Obstetricians (RCOG), and the Royal College of General Practitioners (RCGP) [6–8], advocate for the decriminalisation of abortion in England and Wales, stating that it will remove unnecessary barriers and improve the current clinical practice. Following recent successes in expanding sexual and reproductive rights in the Republic of Ireland and Northern Ireland, there is increasing pressure to decriminalise abortion in England and Wales [9, 10]. This warrants the exploration of new service models which would be available after decriminalisation and could improve current practice.

In low-and-middle-income countries (LMICs), resource constraints motivate policymakers to rethink existing processes and make decisions that are cost effective [11]. Providers then leverage regulatory gaps to adopt “frugal innovations” [12]. Reverse innovation occurs when products/services that are highly effective and scalable penetrate marginalised markets of high-income countries (HICs); and for service models, core elements are extracted and adapted for local conditions [13].

Abortion services have long been part of primary care in LMICs and are sometimes “task-shifted” to mid-level providers (MLPs), such as nurses or midwives. Some studies from LMICs have shown that surgical abortions (SA) can be safely and effectively performed by MLPs in the primary care setting of LMICs [14, 15]. The advent of early medical abortion^1^ (EMA) has further enabled the provision of safe abortion in a simple health facility with few requirements for technology or any surgical skills [17]. Understanding the potential value and challenges of reverse innovation for potential primary care abortion services in the UK is necessary to make strong evidence-based propositions for future policy and legislative changes [18].

In this article, we will:

- systematically review the evidence base for first-trimester abortion services in primary care of LMICs,
- use a narrative synthesis approach to analyse the quality of abortion services with specific indicators organised around the Donabedian model,
- and consider the opportunities and challenges for the development of such services in the UK.

## Methods

This systematic review was conducted using Covidence™ [19], and in accordance with the PRISMA Statement, refer to Additional file 1 [20].

### Search strategy and selection criteria

We searched databases MEDLINE, EMBASE, Global Health, Maternity and Infant Care, Cumulative Index to Nursing and Allied Health Literature (CINAHL) and Health Management Information Consortium (HMIC) (grey literature database) for studies published between September 1994^2^ to February 2020 (Additional file 1: Full search strategy). All searches were limited to papers written in the English language. Other relevant papers were identified by citation searching and reference checking.

We used a PI(C)OS framework to establish search terms and selection criteria (Additional file 1: Search terms and selection criteria) [21]. The population was defined as all healthcare providers strictly in primary care settings, according to the WHO definition^3^ [22]. Studies in secondary and tertiary settings were excluded. NGO-led services were also excluded as they are semi-autonomous and may be separate from formal healthcare systems depending on country. Population was further refined by country, including those classified as “low income,” “lower-middle income,” and “upper-middle income”,^4^ and those where abortion is not entirely prohibited^5^ (Additional file 1: List of LMICs) [23, 24]. The intervention was defined as early abortion (medical or surgical), where “early” implies a pregnancy under 12 weeks of gestation [25]. No comparators were considered as this review is an exploration of existing literature. The outcome was defined as the quality of abortion services, including themes outlined by Dennis et al. shown in Table 1 [26]. Only peer-reviewed primary studies were included. A list of excluded studies can be found in Additional file 1.

**Table 1:** Indicators of high-quality early abortion care explored in this review, adapted from Dennis et al. [26]

Theme	Subtheme	Indicators of high quality
Structure	Law and Policy	•Abortion care must be accessible and not limited by administrative or policy barriers. •Regulations, guidelines and other policy documents have been developed, approved by national/sub-national governments, and/or disseminated to health care facilities that are supportive of access to safe abortion care consistent with WHO guidance.
	Infrastructure	•Efficient, high-quality referral systems are in place. •Essential equipment, supplies and medications are available in sufficient quantity to address needs. •Abortion is provided in a facility with space for privacy.
Process	Technical Competency	•Appropriate pain management techniques are in place. •Physical assessments of general and sexual and reproductive health are performed (including confirmation of gestational age). •Staff follow approved guidelines and protocols for medical, surgical, and incomplete abortion. •Staff use appropriate technologies. •Follow-up care is provided, where client’s experience with abortion and pregnancy status are assessed.
	Information Provision	•Staff explain all aspects of abortion care to clients (current condition, treatment plan, follow-up needs, and potential post-abortion complications and how to obtain appropriate post-abortion care). •Staff provide clients the opportunity to express concerns, ask questions, and receive accurate, understandable answers.
	Client-provider interactions	•Staff offer respectful care. •Staff work to ensure privacy during the visit. •Staff provide confidential care. •Staff hold non-judgemental attitudes. •Staff–client interactions promote an atmosphere of trust.
	Ancillary Services	•Staff directly provide or offer referrals for a range of sexual and reproductive health services, including contraception and screening and treatment for HIV and STIs.
Outcome	Abortion Outcomes	•There is low number of admissions for treatment of abortion complications. •There is a low percentage of maternal deaths as a result of abortion^a^.
	Client Satisfaction	•Clients are satisfied with abortion care

^a^According to WHO in 2008, mortality rate due to unsafe abortion was at 30 deaths per 100,000 live births (13%) worldwide. In developed regions, mortality rate due to unsafe abortion was 0.7 deaths per 100,000 live births (4%); in developing regions, mortality rate due to unsafe abortion was 40 deaths per 100,000 live births (13%) [27]

### Quality appraisal

The quality of papers was assessed using a standardised checklist from the Mixed Methods Appraisal Tool (MMAT) 2018 [28]. This tool was chosen due to the heterogeneity of included papers. An extensive scoring guide and an overall quality score was given for each included study. A detailed assessment of each paper was also conducted, refer to Additional file 1.

### Data synthesis and analysis

A narrative synthesis analysis was used due to the heterogeneity of included studies. JZ extracted the data using a standardised template and summarised the results narratively. RB was involved in identifying relevant themes and reaching a consensus on the data extracted. A thematic analysis was conducted to assess the quality of abortion services according to various quality various indicators at the structural, process, and outcome levels. Table 1 shows the indicators of quality abortion care used in this study.

## Results

**Fig. 1:**
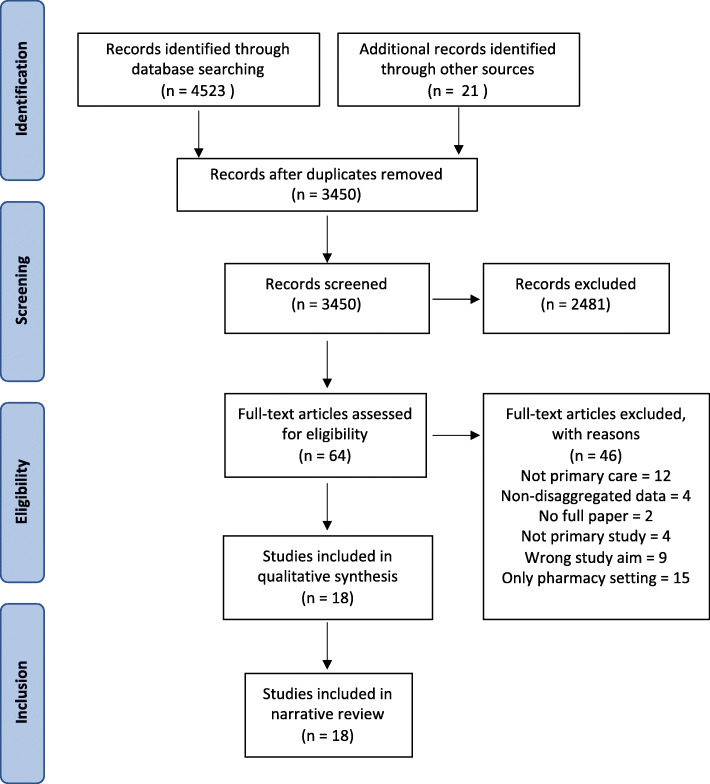
PRISMA Flow Diagram

Figure 1 shows the PRISMA flow diagram of this study [18]. An initial search yielded 3450 titles. Sixty-nine studies were selected for full-text review and an additional 21 studies were identified by forward and backward snowballing. We identified 18 studies for inclusion, of which there were eight implementation studies, three cross-sectional studies, three prospective cohort studies, two qualitative studies and two randomised controlled trials (RCT) (PRISMA diagram in Fig. 1). We describe the included study characteristics in Table 2 (see Additional file 1 for further details). Included studies were conducted in eight countries - Bangladesh [36], Democratic People’s Republic of Korea (DPRK) [45], Ethiopia [30], India [31, 32, 37, 42], Kyrgyzstan [33], Nepal [29, 32, 35, 40, 41, 43, 44, 46], Nigeria [32, 38, 39] and South Africa [34].

**Table 2:** Summary of studies (Additional file 1: Detailed summary table)

First author (Year)	Country	Abortion legality^1^	Provider	Training	Sample size	Study type (Description)	Quality^b^
Andersen et al. (2016) [29]	Nepal	4	ANM^2^	Yes	25,187	**Implementation study** (Site assessments, and quantitative data collected on service provision and quality)	Medium
Assefa et al. (2019) [30]	Ethiopia	4	Nurse, Health Officers, Midwives	No	405	**Cross-sectional study** (Structured questionnaires for MLPs)	High
Banerjee et al. (2010) [31]	India	3	Doctors (Majority OB/GYN^3^)	Yes	60	**Implementation study** (Semi-structured questionnaire at baseline and follow-up, and in-depth interview with doctors)	Medium
Benson et al. (2017) [32]	India, Nepal, Nigeria	3, 4, 1	Physicians, ANM^2^, midwives	Yes	3435	**Prospective cohort study** (Questionnaires for providers at baseline and follow-up over a 4-year period)	Medium
Johnson et al. (2018) [33]	Kyrgyzstan	4	Midwives and family nurses	Yes	554	**Implementation study** (Abortion outcomes of clients, and questionnaires for clients at exit phase)	Medium
Kawonga et al. (2008) [34]	South Africa	4	Did not specify	Yes	290	**Implementation study** (Abortion outcomes of clients and exit interview with clients. Qualitative interviews with 5 providers)	Low
KC NP et al. (2011) [35]	Nepal	4	ANM^2^ and senior nurses	Yes	1799	**Implementation study** (Abortion outcomes of clients and interview on client’s experience; and qualitative site evaluation)	Medium
Marlow et al. (2016) [36]	Bangladesh	1	Did not specify	N/A	10	**Qualitative study** (in-depth interview to understand the perspectives women’s experience on medical abortion)	High
Mundle et al. (2007) [37]	India	3	Doctors	Yes	150	**Implementation study** (Abortion outcome of clients, questionnaires on client experience and daily symptom diary cards for clients)	High
Okonofua et al. (2011) [38]	Nigeria	1	Doctors (Mostly GP^8^ and OB/GYN^3^)	No	122	**Cross-sectional study** (Structured questionnaires for doctors)	High
Okonofua et al. (2005) [39]	Nigeria	1	Doctors (Majority GP^8^ and OB/GYN^3^)	No	323	**Cross-sectional study** (Structured questionnaires for doctors)	High
Puri et al. (2018) [40]	Nepal	4	ANM^2^	Yes	605	**Prospective cohort study (**Structured questionnaire for clients comparing the services in pharmacy vs. public health facility)	Medium
Puri et al. (2014) [41]	Nepal	4	ANM^2^	Yes	241	**Implementation study** (Exit interviews with client, Semi-structured interviews with ANMs)	Low
Ramachandar and Pelto (2005) [42]	India	3	Doctors, nurses, midwives, pharmacists	No	40	**Qualitative study** (in-depth interview with providers on medical abortion)	Medium
Rocca et al. (2018) [43]	Nepal	4	ANM^2^	Yes	605	**Prospective cohort study** (Structured questionnaire for clients comparing the services in pharmacy vs. public health facility)	High
Tamang et al. (2017) [44]	Nepal	4	ANM^2^ + staff nurses (MLP) vs. doctors	Yes	1077	**Randomised controlled trial** (Exit survey “Acceptability form” administered to ascertain women’s experience)	High
Tran et al. (2010) [45]	DPRK	4	Doctors	Yes	199	**Implementation study** (Abortion outcomes of clients and exit survey with clients)	High
Warriner et al. (2011) [46]	Nepal	4	ANM^2^ + staff nurses (MLP) vs. doctors	Yes	1077	**Randomised controlled trial** (Client abortion outcomes)	High

^b^Quality was judged based on MMAT Critical Appraisal Tool – study methods were evaluated against the MMAT checklist, consisting 5 items (Additional file 1). High quality papers met at least 4 criteria; medium quality papers met 2 or 3 criteria; low quality papers met 1 criterion only. Refer to appendix for detailed evaluation of each criteria

1. Abortion legality: (1) To save woman’s life only; (2) To save life, preserve physical and mental health; (3) To save life, preserve physical and mental health, and on socioeconomic grounds; (4) On request; 2. ANM: Auxiliary nurse-midwives; 3. OB/GYN: Obstetrician and Gynaecologists; 4. PCF: Primary care facility; 5. FOP: Felsher Obstetric Points; 6. PHC: Primary health centres; 7. HP: Health posts; 8. GP: General practitioner; 9. RHC: Reproductive health clinics; 10. District hospital is considered as part of its primary care in Nepal

With reference to Dennis et al. [26], we identified a total of 21 indicators to assess the quality of abortion services in eight subthemes, organised under three sections: (1) structural indicators: law and policy, infrastructure; (2) process indicators: technical competency, information provision, client-provider interactions, ancillary services; (3) outcome indicators: abortion outcomes, client satisfaction. The following is a narrative analysis of the abortion services and the contexts in which they operate, detailed in the included studies.

## Structural indicators

### Law and policy

LMICs are disproportionately affected by restrictive abortion laws, therefore unsafe abortions are extremely common in affected countries and result in high maternal mortality rates*.* In response to this phenomenon, the Bangladeshi government sanctioned “menstrual regulation”^6^ (MR), a process to remove the uterine lining using surgical or medical methods, whether the woman is pregnant or not, henceforth enabling one to legally seek help through primary care services [40].

Liberal abortion policies encourage safe abortion services and reduce maternal mortality [47]. Countries (India, Ethiopia, Nepal, Kyrgyzstan, DPRK, South Africa) with liberal abortion law have well-established policies and guidelines for service provision [29–31, 33–35, 40–46]. Nepal and Ethiopia have distinguished themselves from the rest, both adopting proactive and liberal measures to integrate medical abortion (MA) services in their local healthcare system by implementing national guidelines and task-shifting services to MLPs [29, 30, 32, 35, 40, 41, 44, 46] – but the results are vastly different. Nepal became a widely successful case and a regional leader of innovative abortion services while MA services are still lacking in Ethiopia – only 20.5% of providers surveyed have received abortion training, and a majority (71.6%) felt uncomfortable working in a facility that provides abortion due to religious and personal reasons [30]. While a supportive government is necessary to introduce new policies, sociocultural factors such as religion and moral believes can hinder their success.

#### Infrastructure

Andersen et al. [29] reported a third of trained auxiliary nurse-midwives (ANMs) did not provide MA due to the lack of appropriate equipment and medication in Nepal. MA was rarely provided in Nigeria due to the high costs of drugs and tighter restrictions, but clinics were well-equipped to provide SA for incomplete abortions [38]. In South Africa, high medication cost was also a barrier to MA provision [34]. MA combination packs in India and Bangladesh made self-medication safer and more intuitive for women, expanding its access to local pharmacies [36, 37]. Some primary care clinics were unequipped to manage MA complications, but all studies detailed referral systems to secondary or tertiary care [29–46].

Privacy in facilities is essential to creating a safe environment for women, especially in communities where abortions are strongly associated with shame and discrimination. Three studies mentioned the lack of private space as an additional barrier to expanding MA services [34, 40, 44]. Yet, this was only made necessary in cohort studies and RCT studies [29, 32, 34, 35, 40, 41, 43, 44, 46]. No cross-sectional studies mentioned privacy, so its actual practice is lesser known. Only Andersen et al. reported the actual proportion of private rooms in their Nepal study - 71% in primary health centres and 53% in health posts [34].

## Process indicators

### Technical competency

MA is recommended by the WHO as a safe and effective method for abortion in the first trimester [25]. While MA becomes increasingly popular globally, surgical methods remain popular in Nigeria and India due to several reasons. (1) Providers have poor knowledge of MA regimen due to lack of training, i.e. most Nigerian doctors are only familiar with misoprostol as a drug for stomach ulcers as it is not licensed for MA [38, 39]; and India has non-standardised MA treatment as providers often rely on their intuition or personal experience to determine the “correct” dosage and regimen [37]. (2) High cost of medication was a barrier to accessibility, especially in rural areas of India with severe lack of funding [42]. (3) A resistance to change, as providers still prefer surgical methods for being marginally “quicker and easier”, and less prone to complications compared to MA [36, 42]. (4) The burden of responsibility placed on clients to self-manage their MA – hence providers may omit those who are uneducated (who may have difficulty comprehending instructions) and those living faraway (as heavy bleeding starts on the journey back home), as it can cause a greater inconvenience overall [36].

Several studies reported that nurses and midwives gained confidence with MA through ample training and practice, hence improving workflow [29, 32, 33, 35, 40, 41, 43, 44, 46]. Nevertheless, some suggested follow-up interventions to ensure long-term effectiveness, such as provider support networks and follow-up practice assessments [29, 35]. Several studies used an ultrasound scan to confirm gestational age of the pregnancy, but later have considered it unnecessary in most cases, consistent with WHO recommendations [3, 31, 32, 43, 45, 46]. Pain medication was provided to over 90% of clients in most cohort studies and RCT studies [40, 43, 45], but actual practice may be as low as 54%, observed by Banerjee et al. in India [31].

#### Information provision

Ramachandar and Pelto [42] highlighted the importance of effective communication in MA as providers have less control over its outcome, relative to SA. This entails providing accurate and adequate information in a clear and concise manner to manage client expectations and reduce complications [40]. In several studies, providers were trained to brief clients on the procedure, side effects and complications of abortion [29, 31, 33–35, 37, 40, 41, 43–46]. However, actual practice largely depends on provider’s knowledge and communication skills. Banerjee et al. [31] reported that 94% of providers explained the MA procedure to clients, 32% explained the possible side effects, and no providers counselled on complications. Some providers were unsure of what constituted a complication and had various ways of classifying expected effects such as pain and bleeding: “some doctors did not have clear idea of what’s normal bleeding” [42]. Although studies reported a majority of their clients were at least “somewhat prepared” for the procedure in India and Kyrgyzstan, other studies in Nepal and Bangladesh show that some clients experience MA with unaddressed questions [33, 36, 41, 42].

#### Client-provider interaction

Clients in India and Nepal experienced judgement from providers and were treated with disrespect due to the stigma associated with abortion in some contexts [29, 37]. One provider restricted MA by the clients’ social status and education level, believing urban clients can better comprehend the instructions [42]. Since self-medication places a greater emphasis on client knowledge, selectively providing MA may reduce mismanagement, but may inadvertently deprive women in lower social status of MA, especially those in challenging situations, such as aborting without a partner’s or family’s consent. Providers who ensured confidentiality improved client’s trust and comfort during the abortion process. In Nepal, the majority of clients valued the confidential support in clinics as they could receive abortion without informing their family members [41, 44]. Nurses/midwives-led services also improved trust and built strong rapport with women within local communities, as they were posted to each health stations longer than physicians [29, 35].

#### Ancillary services

Provision of contraception may be as low as 42%, reported by Benson et al. across Nigeria, Nepal and India [32]. Short-term contraceptives such as condoms, pills and injectables are popular with clients, whilst long-acting reversible contraceptives (LARC) such as intrauterine devices and implants are less popular [29, 32–44, 46]. One study claimed that given the poor accessibility to health facilities in rural areas, expanding the provision of LARC is important to prevent any unwanted pregnancies [34].

## Outcome indicators

### Abortion outcomes and client satisfaction

The majority of studies showed at least a 95% complete abortion rate, consistent with the international benchmark [29, 33–36, 41, 45, 46, 48]. Only one study failed to meet 95%, attributed to the provider’s lack of experience with MA. All incomplete abortions were either resolved by surgical aspiration in the primary care clinic itself or through a referral to a hospital [29, 33–37, 40, 41, 45, 46]. Most clients were satisfied with the abortion services they received and would recommend them to their friends [33, 35, 42, 45, 46]. Although satisfaction levels were subjective to clients, Tamang et al. [44] showed that high satisfaction rates were related to experiences of shorter length of abortion, a less-than-expected amount of bleeding and high-quality counselling. SA outcomes were not investigated as cross-sectional studies did not allow for patient follow-up [38, 39].

## Discussion

In this review, we explored the quality of first-trimester abortion services provided in primary care clinics of LMICs, using 21 indicators organised around the Donabedian model. In this service model, we observed an efficient workflow that optimised workforce while ensuring safety and client satisfaction. Compared to the classic linear relationship in a Donabedian’s model, our results postulate a mediation pathway where good structure directly promotes good outcome and process, which in turn also promotes good outcome [49]. This pathway underscores the importance of structural components, but our review also showed that policies and infrastructure are insufficient – for example, in countries such as Ethiopia where pro-choice government policies do not necessarily result in accessible abortion services.

Many doctors in Nigeria and India still favoured SA, and some clinics were well-equipped to perform MVA in the event of incomplete abortion. We cannot comment on provider’s competency and knowledge of SA as there was little evidence in the recent literature. As the worldwide trend changes from SA towards MA, our review shows that these components are essential for quality MA services: (1) a safe and private space to ensure client confidentiality for in-clinic abortion, due to its longer duration relative to SA; (2) a standardised understanding of arbitrary side effects, such as bleeding and pain; (3) a strong rapport between clients and providers as EMA focuses on self-medication.

Multiple studies also showed the success of task-shifting – nurses and midwives can effectively replace doctors in abortion services when well-trained and supported. Redistributing low-skilled tasks can optimise efficiency and improve work satisfaction across all providers, thereby combatting healthcare workforce shortages [13]. Passing tasks to midwives and nurses can also build a stronger rapport between provider and clients as they are sometimes able to commit to a local community in the longer-term [35]. Task-shifting is increasingly feasible with the popularity of MA as it requires less technical skills than SA [17]. Shifting the task to well-trained MLPs expands the provider network, thereby increasing service availability.

This review showed that provision of first trimester MA (EMA) in primary care services is safe, feasible and acceptable – decentralising abortion services to primary care and task-shifting will increase availability and accessibility. In the US, studies support the integration of EMA into its primary care system as it essentially uses skills that primary care providers already practice [50, 51]. Some countries, such as Australia, France, and the Netherlands, already provide EMA in primary care clinics [52–54]. In England and Wales, primary care teams in general practice (GPs) or sexual health clinics (SHCs) already provide counselling, pre-abortion screening, and referral into abortion services [55].

Former RCOG president, Anthony Falconer expects the line between primary and secondary women’s healthcare to become fuzzier with more “gynaecological issues” resolved within the community, and the RCOG also proposed a “life-course approach” to women’s health starting in primary care [56, 57]. In recent years, members of parliament across the House of Commons have shown overwhelming support for the decriminalisation of abortion [58, 59]. If this is achieved, EMA services will potentially expand to primary care and align with the NHS Long Term Plan, aimed at facilitating a stronger collaboration between primary and secondary care service for an integrated approach [60]. This expectation necessitates secondary-based trainings for primary healthcare practitioners to ensure technical competency. In RCOG’s 2016 workforce survey of UK consultants, only 5.2% (around 60% of OB/GYN specialists) included abortion as part of their work [61]. Expanding EMA services to GP clinics would increase the number of trained health professionals that perform simple abortion procedures, freeing up specialists for more urgent, complicated cases, such as those seeking abortion in later in pregnancy.

Nevertheless, some challenges need to be addressed while implementing change. Convincing stakeholders of the potential value that “frugal innovations”, such as task-shifting, can bring to the NHS is complex. Innovations from LICs are often discounted or given shorter shrift, and research from these settings is rated worse based on their country of origin [62–64], complicating the diffusion of learning from these contexts. Nonetheless, the extensive experience of primary care EMA in these countries suggests that there is much that could be learned by the UK. There is a risk that introducing EMA into primary care in the UK may increase burden on GP clinics, already face issues of long-waiting hours and workforce shortages. Careful planning would be required to ensure that additional services do not result in a greater inefficiency and cost to the NHS.

### Strengths and limitations of study

Our review had several limitations. First, a disproportionate number of MA papers were included thus less is understood on SA services in primary care due to a paucity of evidence. We also excluded services delivered in NGO clinics as they were not strictly primary healthcare – but are often similar in make-up to primary care clinics and sometimes, the sole providers of abortion services in some LMICs. Second, a majority of included studies were in a controlled environment, where provider practice was standardised by strict protocols – therefore, results may not represent actual practice, and this also reflects a gap in the current literature as more cross-sectional studies should be conducted to give a full picture. Lastly, only two of the 18 studies were qualitative studies, but they contributed more insight in our review as their narrative form provides a deeper understanding of the phenomenon compared to quantitative studies.

## Conclusions

Our review is the first to consolidate quality of abortion services provided in primary care clinics of LMICs. Using the Dennis et al. framework, we determined the components necessary for a successful abortion service in primary care clinics. Overall, we conclude that EMA provision in primary care is safe and feasible, and that implementing a similar service in the UK could improve access without compromising on quality.

The next steps would be a cost estimation of integrating an EMA service into GP clinics, and an economic evaluation to make a strong business proposition. Acceptability and feasibility studies would be required to explore the underlying conditions of primary care EMA. Qualitative studies would also provide an in-depth understanding of attitudes primary care providers and women have towards primary care EMA.

The recent COVID-19 outbreak further builds a strong case for changing policies to match the evidence base. Telemedical EMA was approved in England, Scotland and Wales, so women can now receive EMA at home, via nurse-led telephone consultations and medical abortion packs sent in the post [65]. The temporary approval of this service sets a precedent for abortion-service innovation, and moving forward, we believe implementing EMA in the UK primary care system can complement telemedical services to provide women with face-to-face care in their own community. We also further recommend that further research is conducted to inform and enable task-shifting of first trimester surgical abortions to nurses and midwives in UK primary care.

## Supplementary information

**Additional file 1 Appendix S1.1.** Medline Search Strategy (via Ovid). **Appendix S1.2.** EMBASE Search Strategy (via Ovid). **Appendix S1.3.** Global Health Search Strategy (via Ovid). **Appendix S1.4.** Maternal and Infant Care Search Strategy (via Ovid). **Appendix S1.5**: Health Management Information Consortium Search Strategy (via Ovid). **Appendix S1.6.** CINAHL (via EBSCO). **Appendix S2.** PRISMA Checklist. **Appendix S3.** PI(C)OS Search Terms and Inclusion Criteria. **Appendix S4.** Detailed list of included papers. **Appendix S5.** Exclusion List. **Appendix S6.** Countries sorted on abortion law and income group. **Appendix S7.** Mixed Method Appraisal Tool (Quality Assessment)

## Data Availability

Data used/analysed for this study are available from the corresponding author included in the review.

## Abbreviations

ANMs: Auxiliary nurse-midwives
BPAS: British Pregnancy Advisory Services
DHSC: Department of health and social care
DPRK: Democratic Republic of Korea
EMA: Early medical abortion
FOP: Felsher obstetrics point
GPs: General practitioners
ICPD: International Conference on Population and Development (ICPD)
HICs: High-income countries
HP: Health post
LARCs: Long-acting reversible contraceptives
LMICs: Low-and-middle-income countries
MA: Medical abortion
MR: Menstrual regulation
MVA: Manual vacuum aspiration
MLPs: Mid-level providers
NGOs: Non-governmental organisations
NHS: National Health Services
OB/GYN: Obstetricians and gynaecologists
PCF/PHC: Primary care facility/Primary health clinic
RCGP: Royal College of General Practitioners
RCOG: Royal College of Gynaecologists and Obstetricians
RCT: Randomised controlled trials
RHC: Reproductive health clinics
SA: Surgical abortion
SHCs: Sexual health clinics
WHO: World Health Organisation

## Footnotes

1 WHO recommends 200 mg mifepristone administered orally, followed 1–2 days later by 800 μg misoprostol administered vaginally, sublingually or buccally [16].

2 The chosen cut-off date as the 1994 Cairo International Conference on Population and Development (ICPD) was a turning point for sexual and reproductive health rights. Governments were urged to impose less punitive measures on women seeking abortion, provide safe abortion services and measures to manage complications resulting from unsafe abortions.

3 WHO defines primary care (PC) as the gate keeper to healthcare services and is where “first-contact, accessible, continued, comprehensive and coordinated care” occurs [22].

4 According to the World Bank

5 According to World Abortion Law

6 It was introduced in 1979 as a procedure that regulates a woman’s menstrual cycle when it has been absent for a short period of time. Legally less than 12 weeks from her last menstrual period.
