# Supplementary material for: Systematic review of early abortion services in low- and middle-income country primary care: potential for reverse innovation and application in the UK context
Source: Global Health. 2020 Sep 30;16:91. doi: 10.1186/s12992-020-00613-z (PMC7524570; doi:10.1186/s12992-020-00613-z)
Supplement: Supplementary file 1 — Additional file 1 Appendix S1.1. Medline Search Strategy (via Ovid). Appendix S1.2. EMBASE Search Strategy (via Ovid). Appendix S1.3. Global Health Search Strategy (via Ovid). Appendix S1.4. Maternal and Infant Care Search Strategy (via Ovid). Appendix S1.5: Health Management Information Consortium Search Strategy (via Ovid). Appendix S1.6. CINAHL (via EBSCO). Appendix S2. PRISMA Checklist. Appendix S3. PI(C)OS Search Terms and Inclusion Criteria. Appendix S4. Detailed list of included papers. Appendix S5. Exclusion List. Appendix S6. Countries sorted on abortion law and income group. Appendix S7. Mixed Method Appraisal Tool (Quality Assessment) [file 12992_2020_613_MOESM1_ESM.docx]

**Appendix S1.1: Medline Search Strategy (via Ovid)**

| **#** | **Searches** | **Results** |
| --- | --- | --- |
| 1 | exp Misoprostol/ | 11665 |
| 2 | exp Mifepristone/ | 12598 |
| 3 | exp Abortifacient Agents/ | 175123 |
| 4 | 1 or 2 or 3 | 175123 |
| 5 | (medica* or surgic* or misopristol or mifepristone or abortifac*).mp. [mp=title, abstract, heading word, drug trade name, original title, device manufacturer, drug manufacturer, device trade name, keyword, floating subheading word, candidate term word] | 5184667 |
| 6 | (manual vacuum aspiration or mva).mp. [mp=title, abstract, heading word, drug trade name, original title, device manufacturer, drug manufacturer, device trade name, keyword, floating subheading word, candidate term word] | 7534 |
| 7 | (electric vacuum aspiration or eva).mp. [mp=title, abstract, heading word, drug trade name, original title, device manufacturer, drug manufacturer, device trade name, keyword, floating subheading word, candidate term word] | 3398 |
| 8 | exp "Dilatation and Curettage"/ | 908 |
| 9 | exp Vacuum Curettage/ | 1334 |
| 10 | ("dilation and curettage" or "dilation and evacuation").mp. [mp=title, abstract, heading word, drug trade name, original title, device manufacturer, drug manufacturer, device trade name, keyword, floating subheading word, candidate term word] | 1640 |
| 11 | exp Abortion, Induced/ | 37582 |
| 12 | (abort* or "termina* adj3 pregnan*" or "termina* adj3 baby" or "termina* adj3 f?etus" or menstrual regulation).mp. [mp=title, abstract, heading word, drug trade name, original title, device manufacturer, drug manufacturer, device trade name, keyword, floating subheading word, candidate term word] | 156902 |
| 13 | exp community health workers/ or exp nursing assistants/ or exp physician assistants/ or exp nurses/ or exp nursing staff/ or exp pharmacists/ or exp general practitioners/ or exp physicians, family/ or exp physicians, primary care/ or exp physicians, women/ | 424483 |
| 14 | (health* adj3 worker*).mp. [mp=title, abstract, heading word, drug trade name, original title, device manufacturer, drug manufacturer, device trade name, keyword, floating subheading word, candidate term word] | 59359 |
| 15 | (nurs* or midwi* or mid-wiv*).mp. [mp=title, abstract, heading word, drug trade name, original title, device manufacturer, drug manufacturer, device trade name, keyword, floating subheading word, candidate term word] | 835768 |
| 16 | (mid* adj3 provider*).mp. [mp=title, abstract, heading word, drug trade name, original title, device manufacturer, drug manufacturer, device trade name, keyword, floating subheading word, candidate term word] | 1195 |
| 17 | pharmac*.mp. | 3752046 |
| 18 | exp general practice/ or exp family practice/ or exp primary health care/ | 236842 |
| 19 | afghanistan.mp. or exp Afghanistan/ | 8675 |
| 20 | benin.mp. or exp Benin/ | 5442 |
| 21 | burkina faso.mp. or exp Burkina Faso/ | 5467 |
| 22 | burundi.mp. or exp Burundi/ | 1082 |
| 23 | exp Central African Republic/ or central african republic.mp. | 1377 |
| 24 | chad.mp. or exp Chad/ | 1786 |
| 25 | comoros.mp. or exp Comoros/ | 497 |
| 26 | eritrea.mp. or exp Eritrea/ | 812 |
| 27 | ethiopia.mp. or exp Ethiopia/ | 18626 |
| 28 | gambia.mp. or exp Gambia/ | 3462 |
| 29 | guinea.mp. or exp Guinea/ | 198613 |
| 30 | guinea bissau.mp. or exp Guinea-Bissau/ | 1394 |
| 31 | north korea.mp. or exp "Democratic People's Republic of Korea"/ | 1433 |
| 32 | democratic people republic of korea.mp. [mp=title, abstract, heading word, drug trade name, original title, device manufacturer, drug manufacturer, device trade name, keyword, floating subheading word, candidate term word] | 0 |
| 33 | liberia.mp. or exp Liberia/ | 2330 |
| 34 | malawi.mp. or exp Malawi/ | 8799 |
| 35 | mali.mp. or exp Mali/ | 5113 |
| 36 | mozambique.mp. or exp Mozambique/ | 4644 |
| 37 | nepal.mp. or exp Nepal/ | 13479 |
| 38 | exp Niger/ or niger.mp. | 21835 |
| 39 | rwanda.mp. or exp Rwanda/ | 4193 |
| 40 | somalia.mp. or exp Somalia/ | 2478 |
| 41 | south sudan.mp. or exp South Sudan/ | 649 |
| 42 | syria.mp. or exp Syria/ | 3396 |
| 43 | tajikistan.mp. or exp Tajikistan/ | 1142 |
| 44 | tanzania.mp. or exp Tanzania/ | 17531 |
| 45 | togo.mp. or exp Togo/ | 2051 |
| 46 | uganda.mp. or exp Uganda/ | 21102 |
| 47 | yemen.mp. or exp Yemen/ | 2680 |
| 48 | zimbabwe.mp. or exp Zimbabwe/ | 8166 |
| 49 | 19 or 20 or 21 or 22 or 23 or 24 or 25 or 26 or 27 or 28 or 29 or 30 or 31 or 32 or 33 or 34 or 35 or 36 or 37 or 38 or 39 or 40 or 41 or 42 or 43 or 44 or 45 or 46 or 47 or 48 | 350014 |
| 50 | angola.mp. or exp Angola/ | 1919 |
| 51 | bangladesh.mp. or exp Bangladesh/ | 19523 |
| 52 | exp Bhutan/ or bhutan.mp. | 955 |
| 53 | bolivia.mp. or exp Bolivia/ | 4651 |
| 54 | cape verde.mp. or exp Cabo Verde/ | 625 |
| 55 | cambodia.mp. or exp Cambodia/ | 5977 |
| 56 | cameroon.mp. or exp Cameroon/ | 9045 |
| 57 | cote d ivoire.mp. | 1071 |
| 58 | ivory coast.mp. or exp Cote d'Ivoire/ | 4043 |
| 59 | exp Djibouti/ or djibouti.mp. | 542 |
| 60 | exp "Georgia (Republic)"/ or exp Georgia/ or georgia.mp. | 12945 |
| 61 | ghana.mp. or exp Ghana/ | 13633 |
| 62 | india.mp. or exp India/ | 216664 |
| 63 | indonesia.mp. or exp Indonesia/ | 22864 |
| 64 | kenya.mp. or exp Kenya/ | 25424 |
| 65 | kosovo.mp. or exp Kosovo/ | 1302 |
| 66 | kiribati.mp. | 210 |
| 67 | kyrgyzstan.mp. or exp Kyrgyzstan/ | 1830 |
| 68 | kyrgyz republic.mp. | 161 |
| 69 | lesotho.mp. or exp Lesotho/ | 907 |
| 70 | micronesia.mp. or exp Micronesia/ | 1366 |
| 71 | "federated states of micronesia".mp. | 1051 |
| 72 | moldova.mp. or exp Moldova/ | 1422 |
| 73 | mongolia.mp. or exp Mongolia/ | 5553 |
| 74 | morocco.mp. or exp Morocco/ | 9717 |
| 75 | myanmar.mp. or exp Myanmar/ | 4861 |
| 76 | nigeria.mp. or exp Nigeria/ | 45455 |
| 77 | pakistan.mp. or exp Pakistan/ | 33636 |
| 78 | papua new guinea.mp. or exp Papua New Guinea/ | 7895 |
| 79 | (sao tome and principe).mp. [mp=title, abstract, heading word, drug trade name, original title, device manufacturer, drug manufacturer, device trade name, keyword, floating subheading word, candidate term word] | 196 |
| 80 | atlantic islands/ or exp "sao tome and principe"/ | 984 |
| 81 | solomon islands.mp. or exp Melanesia/ | 1712 |
| 82 | exp Sri Lanka/ or sri lanka.mp. | 10367 |
| 83 | exp Sudan/ or sudan.mp. | 12345 |
| 84 | swaziland.mp. or exp Swaziland/ | 1396 |
| 85 | eswatini.mp. | 75 |
| 86 | timor leste.mp. or exp Timor-Leste/ | 675 |
| 87 | tunisia.mp. or exp Tunisia/ | 12054 |
| 88 | ukraine.mp. or exp Ukraine/ | 18394 |
| 89 | uzbekistan.mp. or exp Uzbekistan/ | 2578 |
| 90 | vanuatu.mp. or exp Vanuatu/ | 741 |
| 91 | vietnam.mp. or exp Vietnam/ | 21670 |
| 92 | zambia.mp. or exp Zambia/ | 7343 |
| 93 | 50 or 51 or 52 or 53 or 54 or 55 or 56 or 57 or 58 or 59 or 60 or 61 or 62 or 63 or 64 or 65 or 66 or 67 or 68 or 69 or 70 or 71 or 72 or 73 or 74 or 75 or 76 or 77 or 78 or 79 or 80 or 81 or 82 or 83 or 84 or 85 or 86 or 87 or 88 or 89 or 90 or 91 or 92 | 508325 |
| 94 | exp Albania/ or albania.mp. | 2136 |
| 95 | american samoa.mp. or exp American Samoa/ | 472 |
| 96 | armenia.mp. or exp Armenia/ | 2398 |
| 97 | azerbaijan.mp. or exp Azerbaijan/ | 2623 |
| 98 | belarus.mp. or exp "Republic of Belarus"/ | 3452 |
| 99 | belize.mp. or exp Belize/ | 1087 |
| 100 | bosnia herzegovina.mp. or exp "Bosnia and Herzegovina"/ | 2836 |
| 101 | botswana.mp. or exp Botswana/ | 3176 |
| 102 | brazil.mp. or exp Brazil/ | 142218 |
| 103 | bulgaria.mp. or exp Bulgaria/ | 11902 |
| 104 | china.mp. or exp China/ | 325450 |
| 105 | colombia.mp. or exp Colombia/ | 23292 |
| 106 | costa rica.mp. or exp Costa Rica/ | 6230 |
| 107 | cuba.mp. or exp Cuba/ | 9070 |
| 108 | dominica.mp. or exp Dominica/ | 695 |
| 109 | equatorial guinea.mp. or exp Equatorial Guinea/ | 654 |
| 110 | ecuador.mp. or exp Ecuador/ | 6679 |
| 111 | fiji.mp. or exp Fiji/ | 2389 |
| 112 | grenada.mp. or exp Grenada/ | 416 |
| 113 | guatemala.mp. or exp Guatemala/ | 5510 |
| 114 | guyana.mp. or exp Guyana/ | 1579 |
| 115 | iran.mp. or exp Iran/ | 65904 |
| 116 | jordan.mp. or exp Jordan/ | 9682 |
| 117 | kazakhstan.mp. or exp Kazakhstan/ | 4695 |
| 118 | lebanon.mp. or exp Lebanon/ | 7154 |
| 119 | exp Libya/ or libya.mp. | 2115 |
| 120 | exp "Macedonia (Republic)"/ or macedonia.mp. | 2258 |
| 121 | malaysia.mp. or exp Malaysia/ | 28362 |
| 122 | maldives.mp. or exp Indian Ocean Islands/ | 5729 |
| 123 | exp Mexico/ or mexico.mp. | 68667 |
| 124 | montenegro.mp. or exp Montenegro/ | 1423 |
| 125 | namibia.mp. or exp Namibia/ | 2044 |
| 126 | nauru.mp. | 188 |
| 127 | paraguay.mp. or exp Paraguay/ | 2192 |
| 128 | peru.mp. or exp Peru/ | 16464 |
| 129 | romania.mp. or exp Romania/ | 16924 |
| 130 | exp Russia/ or russia.mp. | 70705 |
| 131 | exp "Independent State of Samoa"/ or samoa.mp. or exp Samoa/ | 1291 |
| 132 | serbia.mp. or exp Serbia/ | 8145 |
| 133 | south africa.mp. or exp South Africa/ | 61531 |
| 134 | saint lucia.mp. or exp Saint Lucia/ | 165 |
| 135 | exp "Saint Vincent and the Grenadines"/ or saint vincent the grenadines.mp. | 73 |
| 136 | thailand.mp. or exp Thailand/ | 42063 |
| 137 | exp Turkey/ or turkey.mp. | 72322 |
| 138 | turkmenistan.mp. or exp Turkmenistan/ | 775 |
| 139 | tuvalu.mp. | 85 |
| 140 | venezuela.mp. or exp Venezuela/ | 8809 |
| 141 | 94 or 95 or 96 or 97 or 98 or 99 or 100 or 101 or 102 or 103 or 104 or 105 or 106 or 107 or 108 or 109 or 110 or 111 or 112 or 113 or 114 or 115 or 116 or 117 or 118 or 119 or 120 or 121 or 122 or 123 or 124 or 125 or 126 or 127 or 128 or 129 or 130 or 131 or 132 or 133 or 134 or 135 or 136 or 137 or 138 or 139 or 140 | 997054 |
| 142 | 49 or 93 or 141 | 1774570 |
| 143 | 4 or 5 or 6 or 7 or 8 or 9 or 10 | 5342987 |
| 144 | 11 or 12 | 157609 |
| 145 | 13 or 14 or 15 or 16 or 17 or 18 | 4858774 |
| 146 | 142 and 143 and 144 and 145 | 875 |

**Appendix S1.2: EMBASE Search Strategy (via Ovid)**

| **#** | **Searches** | **Results** |
| --- | --- | --- |
| 1 | misoprostol.mp. or exp misoprostol/ or exp mifepristone plus misoprostol/ | 12153 |
| 2 | mifepristone.mp. or exp mifepristone/ | 12968 |
| 3 | (abortifacient* or abortive*).mp. or exp abortive agent/ | 183033 |
| 4 | exp vacuum aspiration/ or "manual vacuum aspiration".mp. or mva.mp. [mp=title, abstract, heading word, drug trade name, original title, device manufacturer, drug manufacturer, device trade name, keyword, floating subheading word, candidate term word] | 8525 |
| 5 | ("electric vacuum aspiration" or eva).mp. [mp=title, abstract, heading word, drug trade name, original title, device manufacturer, drug manufacturer, device trade name, keyword, floating subheading word, candidate term word] | 3398 |
| 6 | exp "dilatation and curettage"/ or "dilation and curettage".mp. or "dilation and evacuation".mp. [mp=title, abstract, heading word, drug trade name, original title, device manufacturer, drug manufacturer, device trade name, keyword, floating subheading word, candidate term word] | 2311 |
| 7 | (medic* or surgic*).mp. [mp=title, abstract, heading word, drug trade name, original title, device manufacturer, drug manufacturer, device trade name, keyword, floating subheading word, candidate term word] | 6192231 |
| 8 | exp surgical abortion/ or exp medical abortion/ | 2146 |
| 9 | (aborti* or "termina* adj3 pregnan*" or "termina* adj3 baby" or "termina* adj3 f?etus" or "menstrual regulation").mp. or exp pregnancy termination/ or exp induced abortion/ or abortion.mp. or exp legal abortion/ | 163276 |
| 10 | exp health care personnel/ | 1579461 |
| 11 | community health worker.mp. or exp health auxiliary/ | 7149 |
| 12 | exp health care personnel/ | 1579461 |
| 13 | exp primary health care/ | 165996 |
| 14 | exp staff nurse/ or exp nurse specialist/ or exp pediatric nurse practitioner/ or exp nurse administrator/ or exp nurse training/ or exp nurse consultant/ or exp expert nurse/ or exp head nurse/ or exp licensed practical nurse/ or exp clinical nurse specialist/ or exp nurse midwife/ or exp nurse practitioner/ or exp adult nurse practitioner/ or exp nurse midwifery/ or exp emergency nurse practitioner/ or exp neonatal nurse practitioner/ or exp family nurse practitioner/ or exp nurse/ or exp practical nurse/ or exp registered nurse/ or exp neonatal nurse/ or exp nurse manager/ | 183060 |
| 15 | exp midwife/ | 32366 |
| 16 | (nurs* or midwi* or mid-wiv*).mp. [mp=title, abstract, heading word, drug trade name, original title, device manufacturer, drug manufacturer, device trade name, keyword, floating subheading word, candidate term word] | 835768 |
| 17 | (health* adj3 worker*).mp. [mp=title, abstract, heading word, drug trade name, original title, device manufacturer, drug manufacturer, device trade name, keyword, floating subheading word, candidate term word] | 59359 |
| 18 | exp general practitioner/ | 101049 |
| 19 | (clinician* or physician* or doctor* or "general practi*").mp. [mp=title, abstract, heading word, drug trade name, original title, device manufacturer, drug manufacturer, device trade name, keyword, floating subheading word, candidate term word] | 1314692 |
| 20 | exp general practice/ | 84146 |
| 21 | exp community pharmacist/ or exp clinical pharmacist/ or exp hospital pharmacist/ or exp pharmacist/ | 76767 |
| 22 | pharmac*.mp. | 3752046 |
| 23 | 1 or 2 or 3 or 4 or 5 or 6 or 7 | 6361775 |
| 24 | 9 and 23 | 55078 |
| 25 | 8 or 24 | 55078 |
| 26 | 10 or 11 or 12 or 13 or 14 or 15 or 16 or 17 or 18 or 19 or 20 or 21 or 22 | 6402910 |
| 27 | afghanistan.mp. or exp Afghanistan/ | 8675 |
| 28 | Benin.mp. or exp Benin/ | 5442 |
| 29 | burundi.mp. or exp Burundi/ | 1082 |
| 30 | Burkina Faso.mp. or exp Burkina Faso/ | 5467 |
| 31 | central african republic.mp. or exp Central African Republic/ | 1377 |
| 32 | chad.mp. or exp Chad/ | 1786 |
| 33 | comoros.mp. or exp Comoros/ | 497 |
| 34 | exp Democratic Republic Congo/ or congo democratic republic.mp. or exp Congo/ | 8020 |
| 35 | eritrea.mp. or exp Eritrea/ | 812 |
| 36 | ethiopia.mp. or exp Ethiopia/ | 18626 |
| 37 | gambia.mp. or exp Gambia/ | 3462 |
| 38 | exp Guinea/ or guinea.mp. | 198613 |
| 39 | guinea bissau.mp. or exp Guinea-Bissau/ | 1394 |
| 40 | north korea.mp. or exp North Korea/ | 1433 |
| 41 | democratic people republic of korea.mp. [mp=title, abstract, heading word, drug trade name, original title, device manufacturer, drug manufacturer, device trade name, keyword, floating subheading word, candidate term word] | 0 |
| 42 | liberia.mp. or exp Liberia/ | 2330 |
| 43 | malawi.mp. or exp Malawi/ | 8799 |
| 44 | mali.mp. or exp Mali/ | 5113 |
| 45 | mozambique.mp. or exp Mozambique/ | 4644 |
| 46 | nepal.mp. or exp Nepal/ | 13479 |
| 47 | exp Niger/ or niger.mp. or exp Niger-Congo people/ | 22195 |
| 48 | rwanda.mp. or exp Rwanda/ | 4193 |
| 49 | somalia.mp. or exp Somalia/ | 2478 |
| 50 | south sudan.mp. or exp South Sudan/ | 649 |
| 51 | syria.mp. or exp Syrian Arab Republic/ | 3396 |
| 52 | tajikistan.mp. or exp Tajikistan/ | 1142 |
| 53 | tanzania.mp. or exp Tanzania/ | 17531 |
| 54 | togo.mp. or exp Togo/ | 2051 |
| 55 | uganda.mp. or exp Uganda/ | 21102 |
| 56 | exp Yemen/ or yemen republic.mp. | 2037 |
| 57 | zimbabwe.mp. or exp Zimbabwe/ | 8166 |
| 58 | 27 or 28 or 29 or 30 or 31 or 32 or 33 or 34 or 35 or 36 or 37 or 38 or 39 or 40 or 41 or 42 or 43 or 44 or 45 or 46 or 47 or 48 or 49 or 50 or 51 or 52 or 53 or 54 or 55 or 56 or 57 | 356512 |
| 59 | angola.mp. or exp Angola/ | 1919 |
| 60 | bangladesh.mp. or exp Bangladesh/ | 19523 |
| 61 | bhutan.mp. or exp Bhutan/ | 955 |
| 62 | bolivia.mp. or exp Bolivia/ | 4651 |
| 63 | cape verde.mp. or exp Cape Verde/ | 625 |
| 64 | cambodia.mp. or exp Cambodia/ | 5977 |
| 65 | cameroon.mp. or exp Cameroon/ | 9045 |
| 66 | cote d ivoire.mp. | 1071 |
| 67 | ivory coast.mp. or exp Cote d'Ivoire/ | 4043 |
| 68 | djibouti.mp. or exp Djibouti/ | 542 |
| 69 | exp "Georgia (republic)"/ or georgia.mp. | 12945 |
| 70 | ghana.mp. or exp Ghana/ | 13633 |
| 71 | exp "Punjab (India)"/ or exp India/ or india.mp. | 216664 |
| 72 | indonesia.mp. or exp Indonesia/ | 22864 |
| 73 | kenya.mp. or exp Kenya/ | 25424 |
| 74 | kosovo.mp. or exp Kosovo/ | 1302 |
| 75 | kiribati.mp. or exp Kiribati/ | 210 |
| 76 | kyrgyz republic.mp. or exp Kyrgyzstan/ | 1695 |
| 77 | kyrgyzstan.mp. | 1830 |
| 78 | lesotho.mp. or exp Lesotho/ | 907 |
| 79 | micronesia.mp. or exp "Federated States of Micronesia"/ | 1366 |
| 80 | moldova.mp. or exp Moldova/ | 1422 |
| 81 | exp Mongolia/ or mongolia.mp. | 5553 |
| 82 | morocco.mp. or exp Morocco/ | 9717 |
| 83 | myanmar.mp. or exp Myanmar/ | 4861 |
| 84 | nigeria.mp. or exp Nigeria/ | 45455 |
| 85 | pakistan.mp. or exp Pakistan/ | 33636 |
| 86 | papua new guinea.mp. or exp Papua New Guinea/ | 7895 |
| 87 | exp "Sao Tome and Principe"/ or sao tome principe.mp. | 88 |
| 88 | exp Solomon Islands/ or solomon islands.mp. | 988 |
| 89 | sri lanka.mp. or exp Sri Lanka/ | 10367 |
| 90 | exp Sudan/ or sudan.mp. | 12345 |
| 91 | swaziland.mp. or exp Swaziland/ | 1396 |
| 92 | eswatini.mp. | 75 |
| 93 | timor leste.mp. or exp Timor-Leste/ | 675 |
| 94 | tunisia.mp. or exp Tunisia/ | 12054 |
| 95 | ukraine.mp. or exp Ukraine/ | 18394 |
| 96 | uzbekistan.mp. or exp Uzbekistan/ | 2578 |
| 97 | vanuatu.mp. or exp Vanuatu/ | 741 |
| 98 | vietnam.mp. or exp Viet Nam/ | 21670 |
| 99 | zambia.mp. or exp Zambia/ | 7343 |
| 100 | 59 or 60 or 61 or 62 or 63 or 64 or 65 or 66 or 67 or 68 or 69 or 70 or 71 or 72 or 73 or 74 or 75 or 76 or 77 or 78 or 79 or 80 or 81 or 82 or 83 or 84 or 85 or 86 or 87 or 88 or 89 or 90 or 91 or 92 or 93 or 94 or 95 or 96 or 97 or 98 or 99 | 507098 |
| 101 | albania.mp. or exp Albania/ | 2136 |
| 102 | american samoa.mp. or exp American Samoa/ | 472 |
| 103 | armenia.mp. or exp Armenia/ | 2398 |
| 104 | azerbaijan.mp. or exp Azerbaijan/ | 2623 |
| 105 | belarus.mp. or exp Belarus/ | 3452 |
| 106 | belize.mp. or exp Belize/ | 1087 |
| 107 | bosnia herzegovina.mp. or exp "Bosnia and Herzegovina"/ | 2836 |
| 108 | botswana.mp. or exp Botswana/ | 3176 |
| 109 | exp "Amazonas (Brazil)"/ or brazil.mp. or exp Brazil/ | 142218 |
| 110 | bulgaria.mp. or exp Bulgaria/ | 11902 |
| 111 | china.mp. or exp China/ | 325450 |
| 112 | colombia.mp. or exp Colombia/ | 23292 |
| 113 | costa rica.mp. or exp Costa Rica/ | 6230 |
| 114 | cuba.mp. or exp Cuba/ | 9070 |
| 115 | exp "Dominican (Dominica)"/ or dominica.mp. or exp Dominica/ | 695 |
| 116 | equatorial guinea.mp. or exp Equatorial Guinea/ | 654 |
| 117 | ecuador.mp. or exp Ecuador/ | 6679 |
| 118 | fiji.mp. or exp Fiji/ | 2389 |
| 119 | grenada.mp. or exp Grenada/ | 416 |
| 120 | guatemala.mp. or exp Guatemala/ | 5510 |
| 121 | guyana.mp. or exp Guyana/ | 1579 |
| 122 | iran.mp. or exp Iran/ | 65904 |
| 123 | jordan.mp. or exp Jordan/ | 9682 |
| 124 | kazakhstan.mp. or exp Kazakhstan/ | 4695 |
| 125 | lebanon.mp. or exp Lebanon/ | 7154 |
| 126 | libya.mp. or exp Libyan Arab Jamahiriya/ | 2115 |
| 127 | exp "Macedonia (republic)"/ or macedonia.mp. | 2258 |
| 128 | malaysia.mp. or exp Malaysia/ | 28362 |
| 129 | maldives.mp. or exp Maldives/ | 433 |
| 130 | exp Mexico/ or exp Mexico City/ or mexico.mp. | 68667 |
| 131 | montenegro.mp. or exp "Montenegro (republic)"/ | 1423 |
| 132 | namibia.mp. or exp Namibia/ | 2044 |
| 133 | nauru.mp. or exp Nauru/ | 188 |
| 134 | paraguay.mp. or exp Paraguay/ | 2192 |
| 135 | peru.mp. or exp Peru/ | 16464 |
| 136 | romania.mp. or exp Romania/ | 16924 |
| 137 | russia.mp. or exp Russian Federation/ | 70705 |
| 138 | russian federation.mp. | 65428 |
| 139 | exp Samoa/ or samoa.mp. | 1291 |
| 140 | exp Serbia/ or serbia.mp. | 8145 |
| 141 | south africa.mp. or exp South Africa/ | 61531 |
| 142 | (saint lucia or st lucia).mp. or exp Saint Lucia/ | 426 |
| 143 | exp "Saint Vincent and the Grenadines"/ or saint vincent the grenadines.mp. | 73 |
| 144 | exp Thailand/ or thailand.mp. | 42063 |
| 145 | turkey.mp. | 72322 |
| 146 | turkmenistan.mp. or exp Turkmenistan/ | 775 |
| 147 | tuvalu.mp. or exp Tuvalu/ | 85 |
| 148 | venezuela.mp. or exp Venezuela/ | 8809 |
| 149 | 101 or 102 or 103 or 104 or 105 or 106 or 107 or 108 or 109 or 110 or 111 or 112 or 113 or 114 or 115 or 116 or 117 or 118 or 119 or 120 or 121 or 122 or 123 or 124 or 125 or 126 or 127 or 128 or 129 or 130 or 131 or 132 or 133 or 134 or 135 or 136 or 137 or 138 or 139 or 140 or 141 or 142 or 143 or 144 or 145 or 146 or 147 or 148 | 994216 |
| 150 | 58 or 100 or 149 | 1777090 |
| 151 | 23 and 24 and 26 and 150 | 1836 |
| 152 | 25 and 26 and 150 | 1836 |

**Appendix S1.3: Global Health Search Strategy (via Ovid)**

| \| **#** \| **Searches** \| **Results** \| \| --- \| --- \| --- \| \| 1 \| misoprostol/ or misoprostol.mp. \| 570 \| \| 2 \| mifepristone.mp. \| 300 \| \| 3 \| abortifacient.mp. or abortifacient properties/ \| 534 \| \| 4 \| (manual vacuum aspiration or mva).mp. [mp=abstract, title, original title, broad terms, heading words, identifiers, cabicodes] \| 725 \| \| 5 \| (electric vacuum aspiration or eva).mp. [mp=abstract, title, original title, broad terms, heading words, identifiers, cabicodes] \| 188 \| \| 6 \| (dilation and curettage).mp. [mp=abstract, title, original title, broad terms, heading words, identifiers, cabicodes] \| 46 \| \| 7 \| (dilation and evacuation).mp. [mp=abstract, title, original title, broad terms, heading words, identifiers, cabicodes] \| 23 \| \| 8 \| abortion/ or exp induced abortion/ or abort*.mp. [mp=abstract, title, original title, broad terms, heading words, identifiers, cabicodes] \| 15024 \| \| 9 \| (termin* adj3 pregnan*).mp. [mp=abstract, title, original title, broad terms, heading words, identifiers, cabicodes] \| 1638 \| \| 10 \| (termin* adj3 f?etus).mp. [mp=abstract, title, original title, broad terms, heading words, identifiers, cabicodes] \| 7 \| \| 11 \| (termin* adj3 baby).mp. [mp=abstract, title, original title, broad terms, heading words, identifiers, cabicodes] \| 2 \| \| 12 \| menstrual regulation.mp. [mp=abstract, title, original title, broad terms, heading words, identifiers, cabicodes] \| 54 \| \| 13 \| (early or first trimester or 1st trimester).mp. [mp=abstract, title, original title, broad terms, heading words, identifiers, cabicodes] \| 176234 \| \| 14 \| exp health care workers/ \| 63052 \| \| 15 \| exp primary health care/ \| 13721 \| \| 16 \| (health* adj3 worker*).mp. [mp=abstract, title, original title, broad terms, heading words, identifiers, cabicodes] \| 42348 \| \| 17 \| (nurs* or midwi* or mid-wiv*).mp. [mp=abstract, title, original title, broad terms, heading words, identifiers, cabicodes] \| 38022 \| \| 18 \| (mid* adj3 provider*).mp. [mp=abstract, title, original title, broad terms, heading words, identifiers, cabicodes] \| 153 \| \| 19 \| pharmac*.mp. \| 236375 \| \| 20 \| (clinician* or physician* or doctor* or "general practi*").mp. [mp=abstract, title, original title, broad terms, heading words, identifiers, cabicodes] \| 82345 \| \| 21 \| 1 or 2 or 3 or 4 or 5 or 6 or 7 \| 2152 \| \| 22 \| (medica* adj3 abortion).mp. [mp=abstract, title, original title, broad terms, heading words, identifiers, cabicodes] \| 365 \| \| 23 \| (surg* adj3 abortion).mp. [mp=abstract, title, original title, broad terms, heading words, identifiers, cabicodes] \| 109 \| \| 24 \| 8 or 9 or 10 or 11 or 12 \| 15909 \| \| 25 \| 21 and 24 \| 907 \| \| 26 \| 22 or 23 or 25 \| 1135 \| \| 27 \| 14 or 15 or 16 or 17 or 18 or 19 or 20 \| 379369 \| \| 28 \| 26 and 27 \| 491 \| \| 29 \| afghanistan.mp. or exp Afghanistan/ \| 2119 \| \| 30 \| benin.mp. or exp Benin/ \| 3324 \| \| 31 \| burkina faso.mp. or exp Burkina Faso/ \| 4480 \| \| 32 \| burundi.mp. or exp Burundi/ \| 727 \| \| 33 \| central african republic.mp. or exp Central African Republic/ \| 1059 \| \| 34 \| exp Chad/ or chad.mp. \| 950 \| \| 35 \| comoros.mp. or exp Comoros/ \| 325 \| \| 36 \| congo democratic republic.mp. or exp Congo Democratic Republic/ \| 4042 \| \| 37 \| exp Eritrea/ or eritrea.mp. \| 375 \| \| 38 \| ethiopia.mp. or exp Ethiopia/ \| 12497 \| \| 39 \| gambia.mp. or exp Gambia/ \| 2625 \| \| 40 \| guinea.mp. or exp Guinea/ \| 15743 \| \| 41 \| guinea-bissau.mp. or exp guinea-bissau/ \| 876 \| \| 42 \| north korea.mp. or exp Korea Democratic People's Republic/ \| 345 \| \| 43 \| liberia.mp. or exp Liberia/ \| 1399 \| \| 44 \| exp Malawi/ or malawi.mp. \| 5826 \| \| 45 \| exp Mali/ or mali.mp. \| 3208 \| \| 46 \| exp Mozambique/ or exp "Gaza (Mozambique)"/ or mozambique.mp. \| 2953 \| \| 47 \| nepal.mp. or exp Nepal/ \| 6907 \| \| 48 \| niger.mp. or exp Niger/ \| 11629 \| \| 49 \| rwanda.mp. or exp Rwanda/ \| 2150 \| \| 50 \| somalia.mp. or exp Somalia/ \| 1304 \| \| 51 \| exp South Sudan/ or south sudan.mp. \| 490 \| \| 52 \| syria.mp. or exp Syria/ \| 1219 \| \| 53 \| tajikistan.mp. or exp Tajikistan/ \| 544 \| \| 54 \| tanzania.mp. or exp Tanzania/ \| 10945 \| \| 55 \| togo.mp. or exp Togo/ \| 1489 \| \| 56 \| uganda.mp. or exp Uganda/ \| 10710 \| \| 57 \| yemen.mp. or exp Yemen/ \| 1546 \| \| 58 \| zimbabwe.mp. or exp Zimbabwe/ \| 4667 \| \| 59 \| 29 or 30 or 31 or 32 or 33 or 34 or 35 or 36 or 37 or 38 or 39 or 40 or 41 or 42 or 43 or 44 or 45 or 46 or 47 or 48 or 49 or 50 or 51 or 52 or 53 or 54 or 55 or 56 or 57 or 58 \| 102765 \| \| 60 \| angola.mp. or exp Angola/ \| 1147 \| \| 61 \| bangladesh.mp. or exp Bangladesh/ \| 11881 \| \| 62 \| bhutan.mp. or exp Bhutan/ \| 571 \| \| 63 \| bolivia.mp. or exp Bolivia/ \| 2501 \| \| 64 \| cape verde.mp. or exp Cape Verde/ \| 226 \| \| 65 \| cambodia.mp. or exp Cambodia/ \| 3084 \| \| 66 \| cameroon.mp. or exp Cameroon/ \| 6458 \| \| 67 \| cote d'ivoire.mp. or exp cote d'ivoire/ \| 4206 \| \| 68 \| ivory coast.mp. \| 4251 \| \| 69 \| djibouti.mp. or exp Djibouti/ \| 331 \| \| 70 \| georgia.mp. or exp "Republic of Georgia"/ or exp Georgia/ \| 5938 \| \| 71 \| ghana.mp. or exp Ghana/ \| 8864 \| \| 72 \| india.mp. or exp India/ or exp "Punjab (India)"/ \| 110823 \| \| 73 \| indonesia.mp. or exp Indonesia/ \| 11075 \| \| 74 \| kenya.mp. or exp Kenya/ \| 15471 \| \| 75 \| kiribati.mp. or exp Kiribati/ \| 142 \| \| 76 \| exp Kosovo/ or kosovo.mp. \| 549 \| \| 77 \| kyrgyz republic.mp. \| 38 \| \| 78 \| kyrgyzstan.mp. or exp Kyrgyzstan/ \| 490 \| \| 79 \| lesotho.mp. or exp Lesotho/ \| 580 \| \| 80 \| exp Micronesia/ or exp "Federated States of Micronesia"/ or micronesia.mp. \| 863 \| \| 81 \| moldova.mp. or exp Moldova/ \| 521 \| \| 82 \| mongolia.mp. or exp Mongolia/ \| 2421 \| \| 83 \| morocco.mp. or exp Morocco/ \| 5889 \| \| 84 \| Myanmar.mp. or exp Myanmar/ \| 3282 \| \| 85 \| nigeria.mp. or exp Nigeria/ \| 30282 \| \| 86 \| pakistan.mp. or exp "Northern Areas (Pakistan)"/ or exp "Federally Administered Tribal Areas (Pakistan)"/ or exp "Punjab (Pakistan)"/ or exp Pakistan/ \| 16125 \| \| 87 \| papua new guinea.mp. or exp Papua New Guinea/ \| 3425 \| \| 88 \| Sao tome principe.mp. or exp "Sao Tome and Principe"/ \| 150 \| \| 89 \| Solomon islands.mp. or exp Solomon Islands/ \| 592 \| \| 90 \| sri lanka.mp. or exp Sri Lanka/ \| 5156 \| \| 91 \| sudan.mp. or exp Sudan/ \| 6006 \| \| 92 \| swaziland.mp. or exp Swaziland/ \| 749 \| \| 93 \| eswatini.mp. [mp=abstract, title, original title, broad terms, heading words, identifiers, cabicodes] \| 30 \| \| 94 \| timor leste.mp. [mp=abstract, title, original title, broad terms, heading words, identifiers, cabicodes] \| 212 \| \| 95 \| eswatini.mp. [mp=abstract, title, original title, broad terms, heading words, identifiers, cabicodes] \| 30 \| \| 96 \| exp east timor/ or exp timor/ \| 321 \| \| 97 \| timor-leste.mp. \| 212 \| \| 98 \| tunisia.mp. or exp Tunisia/ \| 5542 \| \| 99 \| tunisia.mp. or exp Tunisia/ \| 5542 \| \| 100 \| ukraine.mp. or exp Ukraine/ \| 2768 \| \| 101 \| uzbekistan.mp. or exp Uzbekistan/ \| 1131 \| \| 102 \| vanuatu.mp. or exp Vanuatu/ \| 450 \| \| 103 \| vietnam.mp. or exp Vietnam/ \| 8278 \| \| 104 \| zambia.mp. or exp Zambia/ \| 4527 \| \| 105 \| 60 or 61 or 62 or 63 or 64 or 65 or 66 or 67 or 68 or 69 or 70 or 71 or 72 or 73 or 74 or 75 or 76 or 77 or 78 or 79 or 80 or 81 or 82 or 83 or 84 or 85 or 86 or 87 or 88 or 89 or 90 or 91 or 92 or 93 or 94 or 95 or 96 or 97 or 98 or 99 or 100 or 101 or 102 or 103 or 104 \| 263016 \| \| 106 \| albania.mp. or exp Albania/ \| 1042 \| \| 107 \| american samoa.mp. or exp American Samoa/ \| 196 \| \| 108 \| armenia.mp. or exp Armenia/ \| 574 \| \| 109 \| azerbaijan.mp. or exp Azerbaijan/ \| 1120 \| \| 110 \| belarus.mp. or exp Belarus/ \| 996 \| \| 111 \| belize.mp. or exp Belize/ \| 419 \| \| 112 \| (bosnia and herzegovina).mp. [mp=abstract, title, original title, broad terms, heading words, identifiers, cabicodes] \| 758 \| \| 113 \| botswana.mp. or exp Botswana/ \| 1795 \| \| 114 \| exp Brazil/ or brazil.mp. \| 86400 \| \| 115 \| bulgaria.mp. or exp Bulgaria/ \| 4634 \| \| 116 \| china.mp. or exp China/ \| 180041 \| \| 117 \| colombia.mp. or exp Colombia/ \| 9699 \| \| 118 \| costa rica.mp. or exp Costa Rica/ \| 2593 \| \| 119 \| cuba.mp. or exp Cuba/ \| 4580 \| \| 120 \| cuba.mp. or exp Cuba/ \| 4580 \| \| 121 \| exp Dominica/ or dominica.mp. \| 305 \| \| 122 \| equatorial guinea.mp. or exp Equatorial Guinea/ \| 395 \| \| 123 \| ecuador.mp. or exp Ecuador/ \| 2833 \| \| 124 \| exp Fiji/ or fiji.mp. \| 763 \| \| 125 \| grenada.mp. or exp Grenada/ \| 228 \| \| 126 \| guatemala.mp. or exp Guatemala/ \| 2889 \| \| 127 \| guyana.mp. or exp Guyana/ \| 614 \| \| 128 \| iran.mp. or exp Iran/ \| 48245 \| \| 129 \| exp Jordan/ or jordan.mp. \| 3430 \| \| 130 \| kazakhstan.mp. or exp Kazakhstan/ \| 1848 \| \| 131 \| lebanon.mp. or exp Lebanon/ \| 2354 \| \| 132 \| libya.mp. or exp Libya/ \| 998 \| \| 133 \| macedonia.mp. or exp "Republic of Macedonia"/ \| 1144 \| \| 134 \| exp Peninsular Malaysia/ or exp Malaysia/ or malaysia.mp. \| 13205 \| \| 135 \| maldives.mp. or exp Maldives/ \| 191 \| \| 136 \| Mauritius.mp. or exp Mauritius/ \| 602 \| \| 137 \| exp Mexico/ or mexico.mp. \| 23100 \| \| 138 \| exp "Serbia and Montenegro"/ or exp Montenegro/ or Montenegro.mp. \| 849 \| \| 139 \| namibia.mp. or exp Namibia/ \| 984 \| \| 140 \| nauru.mp. or exp Nauru/ \| 89 \| \| 141 \| exp Paraguay River/ or exp Paraguay/ or paraguay.mp. \| 1149 \| \| 142 \| exp Peru/ or peru.mp. \| 7285 \| \| 143 \| romania.mp. or exp Romania/ \| 7723 \| \| 144 \| russia.mp. or exp Russia/ \| 13241 \| \| 145 \| russian federation.mp. \| 11983 \| \| 146 \| exp Samoa/ or samoa.mp. \| 562 \| \| 147 \| exp Serbia/ or serbia.mp. \| 3765 \| \| 148 \| south africa.mp. or exp South Africa/ \| 26351 \| \| 149 \| st lucia.mp. or exp Saint Lucia/ \| 279 \| \| 150 \| exp Saint Vincent/ or saint vincent.mp. \| 112 \| \| 151 \| (Saint Vincent and the grenadines).mp. [mp=abstract, title, original title, broad terms, heading words, identifiers, cabicodes] \| 80 \| \| 152 \| exp "Saint Vincent and the Grenadines"/ or exp Grenadines/ or grenadines.mp. \| 83 \| \| 153 \| exp Thailand/ or thailand.mp. \| 23275 \| \| 154 \| exp Turkey/ or turkey.mp. \| 35923 \| \| 155 \| turkmenistan.mp. or exp Turkmenistan/ \| 410 \| \| 156 \| tuvalu.mp. or exp Tuvalu/ \| 63 \| \| 157 \| venezuela.mp. or exp Venezuela/ \| 5190 \| \| 158 \| 106 or 107 or 108 or 109 or 110 or 111 or 112 or 113 or 114 or 115 or 116 or 117 or 118 or 119 or 120 or 121 or 122 or 123 or 124 or 125 or 126 or 127 or 128 or 129 or 130 or 131 or 132 or 133 or 134 or 135 or 136 or 137 or 138 or 139 or 140 or 141 or 142 or 143 or 144 or 145 or 146 or 147 or 148 or 149 or 150 or 151 or 152 or 153 or 154 or 155 or 156 or 157 \| 500967 \| \| 159 \| 59 or 105 or 158 \| 824983 \| \| 160 \| 28 and 159 \| 317 \| |
| --- | --- | --- | --- | --- | --- | --- | --- | --- | --- | --- | --- | --- | --- | --- | --- | --- | --- | --- | --- | --- | --- | --- | --- | --- | --- | --- | --- | --- | --- | --- | --- | --- | --- | --- | --- | --- | --- | --- | --- | --- | --- | --- | --- | --- | --- | --- | --- | --- | --- | --- | --- | --- | --- | --- | --- | --- | --- | --- | --- | --- | --- | --- | --- | --- | --- | --- | --- | --- | --- | --- | --- | --- | --- | --- | --- | --- | --- | --- | --- | --- | --- | --- | --- | --- | --- | --- | --- | --- | --- | --- | --- | --- | --- | --- | --- | --- | --- | --- | --- | --- | --- | --- | --- | --- | --- | --- | --- | --- | --- | --- | --- | --- | --- | --- | --- | --- | --- | --- | --- | --- | --- | --- | --- | --- | --- | --- | --- | --- | --- | --- | --- | --- | --- | --- | --- | --- | --- | --- | --- | --- | --- | --- | --- | --- | --- | --- | --- | --- | --- | --- | --- | --- | --- | --- | --- | --- | --- | --- | --- | --- | --- | --- | --- | --- | --- | --- | --- | --- | --- | --- | --- | --- | --- | --- | --- | --- | --- | --- | --- | --- | --- | --- | --- | --- | --- | --- | --- | --- | --- | --- | --- | --- | --- | --- | --- | --- | --- | --- | --- | --- | --- | --- | --- | --- | --- | --- | --- | --- | --- | --- | --- | --- | --- | --- | --- | --- | --- | --- | --- | --- | --- | --- | --- | --- | --- | --- | --- | --- | --- | --- | --- | --- | --- | --- | --- | --- | --- | --- | --- | --- | --- | --- | --- | --- | --- | --- | --- | --- | --- | --- | --- | --- | --- | --- | --- | --- | --- | --- | --- | --- | --- | --- | --- | --- | --- | --- | --- | --- | --- | --- | --- | --- | --- | --- | --- | --- | --- | --- | --- | --- | --- | --- | --- | --- | --- | --- | --- | --- | --- | --- | --- | --- | --- | --- | --- | --- | --- | --- | --- | --- | --- | --- | --- | --- | --- | --- | --- | --- | --- | --- | --- | --- | --- | --- | --- | --- | --- | --- | --- | --- | --- | --- | --- | --- | --- | --- | --- | --- | --- | --- | --- | --- | --- | --- | --- | --- | --- | --- | --- | --- | --- | --- | --- | --- | --- | --- | --- | --- | --- | --- | --- | --- | --- | --- | --- | --- | --- | --- | --- | --- | --- | --- | --- | --- | --- | --- | --- | --- | --- | --- | --- | --- | --- | --- | --- | --- | --- | --- | --- | --- | --- | --- | --- | --- | --- | --- | --- | --- | --- | --- | --- | --- | --- | --- | --- | --- | --- | --- | --- | --- | --- | --- | --- | --- | --- | --- | --- | --- | --- | --- | --- | --- | --- | --- | --- | --- | --- | --- | --- | --- | --- | --- | --- | --- | --- | --- | --- | --- | --- | --- | --- | --- | --- | --- | --- | --- | --- | --- | --- | --- | --- | --- | --- | --- | --- | --- | --- | --- | --- | --- | --- | --- | --- | --- | --- | --- | --- | --- | --- | --- | --- | --- | --- | --- | --- | --- | --- | --- | --- | --- | --- | --- | --- | --- | --- | --- | --- | --- | --- | --- | --- | --- | --- |

**Appendix S1.4: Maternal and Infant Care Search Strategy (via Ovid)**

|  | **Searches** | **Results** |
| --- | --- | --- |
| 1 | (Drugs - prescription or Abortifacient agents or Mifepristone or Misoprostol).de. | 1577 |
| 2 | Misoprostol.de. | 366 |
| 3 | Mifepristone.de. | 41 |
| 4 | (medic* or surgic* or misoprostol or mifepristone or abortifac*).mp. [mp=abstract, heading word, title] | 41030 |
| 5 | (manual vacuum aspiration or mva).mp. [mp=abstract, heading word, title] | 44 |
| 6 | (electric vacuum aspiration or eva).mp. [mp=abstract, heading word, title] | 9 |
| 7 | (dilation and curettage).mp. [mp=abstract, heading word, title] | 54 |
| 8 | (dilation and evacuation).mp. [mp=abstract, heading word, title] | 30 |
| 9 | ("Dilatation and curettage" or "Misoprostol - therapeutic use").de. | 126 |
| 10 | Surgical procedures - operative.de. | 266 |
| 11 | Abortion.de. | 36 |
| 12 | Induced abortion.de. | 2 |
| 13 | (abort* or "termina* adj3 pregnan*" or "termina* adj3 baby" or "termina* adj3 f?etus" or menstrual regulation).mp. [mp=abstract, heading word, title] | 6224 |
| 14 | 1 or 2 or 3 or 4 or 5 or 6 or 7 or 8 or 9 or 10 | 41814 |
| 15 | 11 or 12 or 13 | 6224 |
| 16 | 14 and 15 | 1362 |
| 17 | (Maternity support workers or Maternal health services or Support workers or Maternity care assistants).de. | 7907 |
| 18 | (Midwifery or Emergency medical services or Voluntary workers).de. | 7504 |
| 19 | Physicians.de. | 76 |
| 20 | (General practitioners or "Quality of health care").de. | 2350 |
| 21 | (General practice or Nursing).de. | 1885 |
| 22 | (Midwives or Nurses).de. | 4971 |
| 23 | (Health care assistants or "Role (health care assistants)" or "Role (midwives)" or Midwives or Health care assistants or Maternal health services or Midwifery or Maternity care assistants).de. | 19975 |
| 24 | (health* adj3 worker*).mp. [mp=abstract, heading word, title] | 2382 |
| 25 | (nurs* or midwi* or mid-wiv*).mp. [mp=abstract, heading word, title] | 48233 |
| 26 | (mid* adj3 provider*).mp. [mp=abstract, heading word, title] | 154 |
| 27 | (Contraceptives - postcoital or Pharmacies or Drugs - prescription or Pharmacists).de. | 1341 |
| 28 | pharmac*.mp. [mp=abstract, heading word, title] | 3443 |
| 29 | ("Knowledge (doctors)" or "Attitudes (doctors)" or Family-centred care or "Attitudes (family physicians)" or "Clinical practice (doctors)").de. | 678 |
| 30 | Family physicians.de. | 49 |
| 31 | (clinician* or physician* or doctor* or "general practi*").mp. [mp=abstract, heading word, title] | 15945 |
| 32 | 17 or 18 or 19 or 20 or 21 or 22 or 23 or 24 or 25 or 26 or 27 or 28 or 29 or 30 or 31 | 69597 |
| 33 | 16 and 32 | 337 |
| 34 | Afghanistan.de. or afghanistan.mp. | 203 |
| 35 | Benin.de. or benin.mp. | 158 |
| 36 | Burkina Faso.de. or burkina faso.mp. | 273 |
| 37 | Burundi.de. or burundi.mp. | 35 |
| 38 | Central African Republic.de. or central african republic.mp. | 17 |
| 39 | Chad.de. or chad.mp. | 43 |
| 40 | comoros.mp. [mp=abstract, heading word, title] | 5 |
| 41 | congo democratic republic.mp. or "Democratic Republic of Congo".de. | 23 |
| 42 | Eritrea.de. or eritrea.mp. | 44 |
| 43 | Ethiopia.de. or ethiopia.mp. | 946 |
| 44 | Gambia.de. or gambia.mp. | 164 |
| 45 | Guinea.de. or guinea.mp. | 302 |
| 46 | Guinea-Bissau.de. or guinea bissau.mp. | 97 |
| 47 | north korea.mp. | 3 |
| 48 | Liberia.de. or liberia.mp. | 69 |
| 49 | Malawi.de. or malawi.mp. | 604 |
| 50 | mali.mp. or Mali.de. | 128 |
| 51 | Mozambique.de. or mozambique.mp. | 202 |
| 52 | Nepal.de. or nepal.mp. | 605 |
| 53 | niger.mp. or Niger.de. | 111 |
| 54 | Rwanda.de. | 35 |
| 55 | Somalia.de. or somalia.mp. | 94 |
| 56 | south sudan.mp. | 43 |
| 57 | Syria.de. or syria.mp. | 42 |
| 58 | Tajikistan.de. or tajikistan.mp. | 16 |
| 59 | Tanzania.de. or tanzania.mp. | 872 |
| 60 | togo.mp. or Togo.de. | 24 |
| 61 | uganda.mp. or Uganda.de. | 778 |
| 62 | Yemen.de. or yemen.mp. | 69 |
| 63 | zimbabwe.mp. or Zimbabwe.de. | 352 |
| 64 | 34 or 35 or 36 or 37 or 38 or 39 or 40 or 41 or 42 or 43 or 44 or 45 or 46 or 47 or 48 or 49 or 50 or 51 or 52 or 53 or 54 or 55 or 56 or 57 or 58 or 59 or 60 or 61 or 62 or 63 | 5600 |
| 65 | Angola.de. or angola.mp. | 47 |
| 66 | Bangladesh.de. or bangladesh.mp. | 866 |
| 67 | Bhutan.de. or bhutan.mp. | 27 |
| 68 | Bolivia.de. or bolivia.mp. | 148 |
| 69 | (cape town or cape verde).mp. [mp=abstract, heading word, title] | 204 |
| 70 | Cambodia.de. or cambodia.mp. | 143 |
| 71 | Cameroon.de. or cameroon.mp. | 176 |
| 72 | Ivory Coast.de. or ivory coast.mp. | 21 |
| 73 | Cote d'Ivoire.de. or cote divoire.mp. | 48 |
| 74 | Djibouti.de. or djibouti.mp. | 9 |
| 75 | Georgia.de. or georgia.mp. | 236 |
| 76 | Ghana.de. or ghana.mp. | 745 |
| 77 | India.de. or india.mp. | 2140 |
| 78 | Indonesia.de. or indonesia.mp. | 436 |
| 79 | Kenya.de. or kenya.mp. | 983 |
| 80 | kiribati.mp. [mp=abstract, heading word, title] | 8 |
| 81 | kosovo.mp. or Kosovo.de. | 18 |
| 82 | kyrgyz republic.mp. [mp=abstract, heading word, title] | 5 |
| 83 | Kyrgyzstan.de. or kyrgyzstan.mp. | 14 |
| 84 | Lesotho.de. or lesotho.mp. | 34 |
| 85 | Micronesia.de. or micronesia.mp. | 10 |
| 86 | moldova.mp. or Moldova.de. | 23 |
| 87 | Mongolia.de. or mongolia.mp. | 45 |
| 88 | Morocco.de. or morocco.mp. | 69 |
| 89 | myanmar.mp. or Myanmar.de. | 108 |
| 90 | Nigeria.de. or nigeria.mp. | 1524 |
| 91 | pakistan.mp. or Pakistan.de. | 638 |
| 92 | papua new guinea.mp. or Papua New Guinea.de. | 121 |
| 93 | sao tome principe.mp. [mp=abstract, heading word, title] | 0 |
| 94 | solomon islands.mp. [mp=abstract, heading word, title] | 13 |
| 95 | Sri Lanka.de. or sri lanka.mp. | 179 |
| 96 | Sudan.de. or sudan.mp. | 254 |
| 97 | swaziland.mp. or Swaziland.de. | 53 |
| 98 | eswatini.mp. [mp=abstract, heading word, title] | 5 |
| 99 | timor*.mp. or East Timor.de. | 42 |
| 100 | Tunisia.de. or tunisia.mp. | 42 |
| 101 | ukraine.mp. or Ukraine.de. | 94 |
| 102 | Uzbekistan.de. or uzbekistan.mp. | 18 |
| 103 | vanuatu.mp. or Vanuatu.de. | 13 |
| 104 | vietnam.mp. or Vietnam.de. | 309 |
| 105 | Zambia.de. or zambia.mp. | 430 |
| 106 | 65 or 66 or 67 or 68 or 69 or 70 or 71 or 72 or 73 or 74 or 75 or 76 or 77 or 78 or 79 or 80 or 81 or 82 or 83 or 84 or 85 or 86 or 87 or 88 or 89 or 90 or 91 or 92 or 93 or 94 or 95 or 96 or 97 or 98 or 99 or 100 or 101 or 102 or 103 or 104 or 105 | 9254 |
| 107 | Albania.de. or albania.mp. | 31 |
| 108 | american samoa.mp. [mp=abstract, heading word, title] | 5 |
| 109 | Armenia.de. or armenia.mp. | 31 |
| 110 | Azerbaijan.de. or azerbaijan.mp. | 27 |
| 111 | belarus.mp. or Belarus.de. | 39 |
| 112 | belize.mp. or Belize.de. | 12 |
| 113 | Bosnia.de. or bosnia.mp. | 36 |
| 114 | Bosnia-Herzegovina.de. or herzegovina.mp. | 29 |
| 115 | Botswana.de. or botswana.mp. | 108 |
| 116 | Brazil.de. or brazil.mp. | 1252 |
| 117 | bulgaria.mp. or Bulgaria.de. | 35 |
| 118 | china.mp. or China.de. | 1988 |
| 119 | colombia.mp. or Colombia.de. | 164 |
| 120 | costa rica.mp. or Costa Rica.de. | 51 |
| 121 | Cuba.de. or cuba.mp. | 56 |
| 122 | Dominica.mp. [mp=abstract, heading word, title] | 2 |
| 123 | equatorial guinea.mp. [mp=abstract, heading word, title] | 5 |
| 124 | Ecuador.de. or ecuador.mp. | 107 |
| 125 | Guatemala.de. or guatemala.mp. | 270 |
| 126 | Guyana.de. or guyana.mp. | 16 |
| 127 | Iran.de. or iran.mp. | 502 |
| 128 | Jordan.de. or jordan.mp. | 209 |
| 129 | Kazakhstan.de. or kazakhstan.mp. | 39 |
| 130 | lebanon.mp. or Lebanon.de. | 141 |
| 131 | Libya.de. or libya.mp. | 13 |
| 132 | macedonia.mp. or "Macedonia (Republic)".de. | 11 |
| 133 | malaysia.mp. or Malaysia.de. | 234 |
| 134 | Maldives.de. or maldives.mp. | 13 |
| 135 | Mauritius.de. or mauritius.mp. | 15 |
| 136 | Mexico.de. or mexico.mp. | 699 |
| 137 | montenegro.mp. [mp=abstract, heading word, title] | 3 |
| 138 | Namibia.de. or namibia.mp. | 52 |
| 139 | nauru.mp. [mp=abstract, heading word, title] | 0 |
| 140 | Paraguay.de. or paraguay.mp. | 20 |
| 141 | peru.mp. or Peru.de. | 253 |
| 142 | Romania.de. or romania.mp. | 79 |
| 143 | russia*.mp. or Russia.de. | 236 |
| 144 | samoa.mp. or Samoa.de. | 23 |
| 145 | serbia.mp. or Serbia.de. | 32 |
| 146 | South Africa.de. or south africa.mp. | 1445 |
| 147 | (saint lucia or st lucia).mp. [mp=abstract, heading word, title] | 6 |
| 148 | saint vincent.mp. [mp=abstract, heading word, title] | 4 |
| 149 | grenadines.mp. [mp=abstract, heading word, title] | 2 |
| 150 | Thailand.de. or thailand.mp. | 467 |
| 151 | turkey.mp. or Turkey.de. | 574 |
| 152 | Turkmenistan.de. or turkmenistan.mp. | 8 |
| 153 | tuvalu.mp. [mp=abstract, heading word, title] | 3 |
| 154 | Venezuela.de. or venezuela.mp. | 32 |
| 155 | 107 or 108 or 109 or 110 or 111 or 112 or 113 or 114 or 115 or 116 or 117 or 118 or 119 or 120 or 121 or 122 or 123 or 124 or 125 or 126 or 127 or 128 or 129 or 130 or 131 or 132 or 133 or 134 or 135 or 136 or 137 or 138 or 139 or 140 or 141 or 142 or 143 or 144 or 145 or 146 or 147 or 148 or 149 or 150 or 151 or 152 or 153 or 154 | 8670 |
| 156 | 64 or 106 or 155 | 21771 |
| 157 | 33 and 156 | 79 |

**Appendix S1.5: Health Management Information Consortium Search Strategy (via Ovid)**

| **#** | **Searches** | **Results** |
| --- | --- | --- |
| 1 | exp abortion/ or exp abortifacients/ or exp abortion services/ | 643 |
| 2 | (abort* or "termin* adj3 pregnancy*" or "termin* adj3 baby*" or "termin* adj3 f?etus" or "menstrual regulation").mp. [mp=title, other title, abstract, heading words] | 958 |
| 3 | (misoprostol or mifepristone).mp. [mp=title, other title, abstract, heading words] | 22 |
| 4 | exp Surgical procedures/ | 92 |
| 5 | ("manual vacuum aspiration" or "electric vacuum aspiration" or "mva" or "eva").mp. [mp=title, other title, abstract, heading words] | 12 |
| 6 | (dilation and curettage).mp. [mp=title, other title, abstract, heading words] | 1 |
| 7 | (dilation and evacuation).mp. [mp=title, other title, abstract, heading words] | 1 |
| 8 | (first trimester or 1st trimester or early).mp. [mp=title, other title, abstract, heading words] | 10495 |
| 9 | exp primary care/ or exp health service staff/ or exp Health professionals/ | 72525 |
| 10 | exp general practitioners/ or exp family practitioners/ or exp general practice staff/ or exp medical staff/ or exp general practice/ or exp general practice profession/ or exp home visits by doctor/ or exp local health care cooperatives/ or exp local health groups/ or exp local medical committees/ or exp primary care groups/ | 31954 |
| 11 | (health* adj3 worker).mp. [mp=title, other title, abstract, heading words] | 428 |
| 12 | (mid* adj3 provider*).mp. [mp=title, other title, abstract, heading words] | 22 |
| 13 | exp Registered general nurses/ or exp Private nurses/ or exp State enrolled nurses/ or exp Named nurses/ or exp Agency nurses/ or exp Intensive care nurses/ or exp Accident & emergency nurses/ or exp Night nurses/ or exp Overseas nurses/ or exp Nurses/ or exp Charge nurses/ or exp Senior nurses/ or exp Occupational health nurses/ or exp Attached general practice nurses/ or exp Community nurses/ or exp district nurses/ or exp Staff nurses/ or exp general practice nurses/ or exp Liaison nurses/ or exp Auxiliary nurses/ or exp Regional nurses/ or exp nursery nurses/ or exp Residential nurses/ or exp Voluntary nurses/ or exp Contact nurses/ or exp State registered nurses/ or exp Primary care nurses/ or exp Cadet nurses/ | 21058 |
| 14 | exp Midwives/ or exp Community midwives/ or exp Staff midwives/ or exp Named midwives/ | 2270 |
| 15 | (nurs* or midwi* or mid-wiv*).mp. [mp=title, other title, abstract, heading words] | 48657 |
| 16 | exp Community pharmacists/ or exp Clinical pharmacists/ or exp Pharmacists/ or exp Hospital pharmacists/ | 2202 |
| 17 | exp Pharmacies/ or pharmac*.mp. or exp Drug dispensing/ | 11556 |
| 18 | (clinician* or physician* or doctor* or "general practi*").mp. [mp=title, other title, abstract, heading words] | 47174 |
| 19 | exp community health workers/ or exp community workers/ or exp community health care/ | 5916 |
| 20 | exp Pharmacy assistants/ or exp Clinical assistants/ or exp Health care assistants/ or exp Care assistants/ or exp Nursing assistants/ | 987 |
| 21 | 1 or 2 or 3 or 4 or 5 or 6 or 7 | 1071 |
| 22 | 9 or 10 or 11 or 12 or 13 or 14 or 15 or 16 or 17 or 18 or 19 or 20 | 122237 |
| 23 | 21 and 22 | 319 |
| 24 | afghanistan.mp. or exp Afghanistan/ | 64 |
| 25 | benin.mp. | 3 |
| 26 | burkina.mp. or exp Burkina Faso/ | 12 |
| 27 | burundi.mp. | 1 |
| 28 | Central African republic.mp. or exp Central African Republic/ | 2 |
| 29 | chad.mp. or exp Chad/ | 3 |
| 30 | comoros.mp. | 1 |
| 31 | congo democratic republic.mp. | 2 |
| 32 | eritrea.mp. or exp Eritrea/ | 2 |
| 33 | ethiopia.mp. or exp Ethiopia/ | 30 |
| 34 | gambia.mp. or exp Gambia/ | 15 |
| 35 | exp Guinea/ or guinea.mp. | 60 |
| 36 | guinea bissau.mp. or exp Guinea Bissau/ | 6 |
| 37 | north korea.mp. or exp North Korea/ | 5 |
| 38 | Liberia.mp. or exp Liberia/ | 12 |
| 39 | Malawi.mp. or exp Malawi/ | 27 |
| 40 | mali.mp. or exp Mali/ | 12 |
| 41 | mozambique.mp. or exp Mozambique/ | 23 |
| 42 | nepal.mp. or exp Nepal/ | 38 |
| 43 | niger.mp. or exp Niger/ | 12 |
| 44 | rwanda.mp. or exp Rwanda/ | 11 |
| 45 | somalia.mp. or exp Somalia/ | 24 |
| 46 | south sudan.mp. | 2 |
| 47 | syria.mp. or exp Syria/ | 18 |
| 48 | tajikstan.mp. | 1 |
| 49 | tanzania.mp. or exp Tanzania/ | 48 |
| 50 | togo.mp. | 2 |
| 51 | uganda.mp. or exp Uganda/ | 66 |
| 52 | yemen.mp. or exp Yemen/ | 13 |
| 53 | zimbabwe.mp. or exp Zimbabwe/ | 75 |
| 54 | 24 or 25 or 26 or 27 or 28 or 29 or 30 or 31 or 32 or 33 or 34 or 35 or 36 or 37 or 38 or 39 or 40 or 41 or 42 or 43 or 44 or 45 or 46 or 47 or 48 or 49 or 50 or 51 or 52 or 53 | 484 |
| 55 | angola.mp. or exp Angola/ | 12 |
| 56 | bangladesh.mp. or exp Bangladesh/ | 100 |
| 57 | bhutan.mp. or exp Bhutan/ | 4 |
| 58 | bolivia.mp. or exp Bolivia/ | 20 |
| 59 | exp Cape Town/ or cape.mp. | 61 |
| 60 | cambodia.mp. or exp Cambodia/ | 17 |
| 61 | cameroon.mp. or exp Cameroun/ | 14 |
| 62 | cote divoire.mp. or exp Ivory Coast/ | 5 |
| 63 | djibouti.mp. or exp Djibouti/ | 4 |
| 64 | exp Georgia Europe/ or georgia.mp. or exp Georgia/ | 103 |
| 65 | ghana.mp. or exp Ghana/ | 56 |
| 66 | india.mp. or exp India/ | 462 |
| 67 | indonesia.mp. or exp Indonesia/ | 83 |
| 68 | kenya.mp. or exp Kenya/ | 75 |
| 69 | Kiribati.mp. [mp=title, other title, abstract, heading words] | 0 |
| 70 | kosovo.mp. or exp Kosovo/ | 16 |
| 71 | Kyrgyz republic.mp. | 1 |
| 72 | kyrgyzstan.mp. or exp Kyrgyzstan/ | 27 |
| 73 | lesotho.mp. or exp Lesotho/ | 15 |
| 74 | micronesia.mp. | 3 |
| 75 | moldova.mp. or exp Moldova/ | 28 |
| 76 | mongolia.mp. or exp Mongolia/ | 11 |
| 77 | morocco.mp. or exp Morocco/ | 29 |
| 78 | myanmar.mp. or exp Myanmar/ | 5 |
| 79 | nigeria.mp. or exp Nigeria/ | 102 |
| 80 | pakistan.mp. or exp Pakistan/ | 105 |
| 81 | papua new guinea.mp. or exp Papua New Guinea/ | 18 |
| 82 | sao tome principe.mp. [mp=title, other title, abstract, heading words] | 0 |
| 83 | Solomon islands.mp. [mp=title, other title, abstract, heading words] | 0 |
| 84 | Sri Lanka.mp. or exp Sri Lanka/ | 58 |
| 85 | sudan.mp. or exp Sudan/ | 33 |
| 86 | swaziland.mp. or exp Swaziland/ | 11 |
| 87 | eswatini.mp. [mp=title, other title, abstract, heading words] | 0 |
| 88 | timor leste.mp. | 4 |
| 89 | tunisia.mp. or exp Tunisia/ | 26 |
| 90 | ukraine.mp. or exp Ukraine/ | 56 |
| 91 | uzbekistan.mp. or exp Uzbekistan/ | 25 |
| 92 | vanuatu.mp. or exp Vanuatu/ | 1 |
| 93 | vietnam.mp. or exp Vietnam/ | 115 |
| 94 | zambia.mp. or exp Zambia/ | 42 |
| 95 | 55 or 56 or 57 or 58 or 59 or 60 or 61 or 62 or 63 or 64 or 65 or 66 or 67 or 68 or 69 or 70 or 71 or 72 or 73 or 74 or 75 or 76 or 77 or 78 or 79 or 80 or 81 or 82 or 83 or 84 or 85 or 86 or 87 or 88 or 89 or 90 or 91 or 92 or 93 or 94 | 1347 |
| 96 | albania.mp. or exp Albania/ | 32 |
| 97 | american samoa.mp. | 2 |
| 98 | armenia.mp. or exp Armenia/ | 25 |
| 99 | azerbaijan.mp. or exp Azerbaijan/ | 18 |
| 100 | belarus.mp. or exp Belarus/ | 34 |
| 101 | belize.mp. or exp Belize/ | 4 |
| 102 | Bosnia Herzegovina.mp. or exp Bosnia Herzegovina/ | 17 |
| 103 | botswana.mp. or exp Botswana/ | 27 |
| 104 | brazil.mp. or exp Brazil/ | 253 |
| 105 | bulgaria.mp. or exp Bulgaria/ | 97 |
| 106 | exp China/ or china.mp. | 911 |
| 107 | colombia.mp. or exp Colombia/ | 49 |
| 108 | costa rica.mp. or exp Costa Rica/ | 36 |
| 109 | cuba.mp. or exp Cuba/ | 52 |
| 110 | dominica.mp. | 1 |
| 111 | equatorial guinea.mp. or exp Equatorial Guinea/ | 1 |
| 112 | ecuador.mp. or exp Ecuador/ | 18 |
| 113 | fiji.mp. or exp Fiji/ | 5 |
| 114 | grenada.mp. | 1 |
| 115 | guatemala.mp. or exp Guatemala/ | 11 |
| 116 | guyana.mp. or exp Guyana/ | 4 |
| 117 | iran.mp. or exp Iran/ | 66 |
| 118 | jordan.mp. or exp Jordan/ | 52 |
| 119 | kazakhstan.mp. or exp Kazakhstan/ | 38 |
| 120 | lebanon.mp. or exp Lebanon/ | 28 |
| 121 | libya.mp. or exp Libya/ | 9 |
| 122 | macedonia.mp. or exp FYR Macedonia/ | 21 |
| 123 | malaysia.mp. or exp Malaysia/ | 83 |
| 124 | maldives.mp. | 3 |
| 125 | mauritius.mp. or exp Mauritius/ | 9 |
| 126 | exp Mexico/ or mexico.mp. | 236 |
| 127 | montenegro.mp. or exp Montenegro/ | 12 |
| 128 | namibia.mp. or exp Namibia/ | 6 |
| 129 | nauru.mp. | 2 |
| 130 | paraguay.mp. or exp Paraguay/ | 6 |
| 131 | peru.mp. or exp Peru/ | 49 |
| 132 | romania.mp. or exp Romania/ | 137 |
| 133 | russia.mp. or exp Russia/ | 285 |
| 134 | russian federation.mp. or exp Soviet Union/ | 188 |
| 135 | samoa.mp. or exp Samoa/ | 6 |
| 136 | serbia.mp. or exp Serbia/ | 36 |
| 137 | south africa.mp. or exp South Africa/ | 351 |
| 138 | st lucia.mp. | 2 |
| 139 | saint lucia.mp. | 1 |
| 140 | diabetes services/ | 220 |
| 141 | (Saint Vincent and the grenadines).mp. [mp=title, other title, abstract, heading words] | 0 |
| 142 | thailand.mp. or exp Thailand/ | 148 |
| 143 | turkey.mp. or exp Turkey/ | 200 |
| 144 | turkmenistan.mp. or exp Turkmenistan/ | 24 |
| 145 | tuvalu.mp. [mp=title, other title, abstract, heading words] | 0 |
| 146 | venezuela.mp. or exp Venezuela/ | 23 |
| 147 | 96 or 97 or 98 or 99 or 100 or 101 or 102 or 103 or 104 or 105 or 106 or 107 or 108 or 109 or 110 or 111 or 112 or 113 or 114 or 115 or 116 or 117 or 118 or 119 or 120 or 121 or 122 or 123 or 124 or 125 or 126 or 127 or 128 or 129 or 130 or 131 or 132 or 133 or 134 or 135 or 136 or 137 or 138 or 139 or 140 or 141 or 142 or 143 or 144 or 145 or 146 | 3048 |
| 148 | 54 or 95 or 147 | 4297 |
| 149 | 23 and 148 | 5 |

**Appendix S1.6: CINAHL (via EBSCO)**

| # | **Search Terms** | **Results** |
| --- | --- | --- |
| S87 | "georgia" | (9,374) |
| S86 | (MH "Djibouti") | (47) |
| S85 | (MH "Cote d'Ivoire") OR "côte d'ivoire or ivory coast" | (299) |
| S84 | (MH "Cameroon") | (1,095) |
| S83 | "cambodia" | (1,612) |
| S82 | (MH "Cape Verde") | (27) |
| S81 | (MH "Bolivia") | (556) |
| S80 | (MH "Bhutan") | (193) |
| S79 | (MH "Bangladesh") | (3,739) |
| S78 | (MH "Angola") | (250) |
| S77 | S47 OR S48 OR S49 OR S50 OR S51 OR S52 OR S53 OR S54 OR S55 OR S56 OR S57 OR S58 OR S59 OR S60 OR S61 OR S62 OR S63 OR S64 OR S65 OR S66 OR S67 OR S68 OR S69 OR S70 OR S71 OR S72 OR S73 OR S74 OR S75 OR S76 | (48,960) |
| S76 | "zimbabwe" | (1,734) |
| S75 | (MH "Yemen") | (430) |
| S74 | (MH "Uganda") | (4,418) |
| S73 | (MH "Togo") | (142) |
| S72 | (MH "Tanzania") | (3,288) |
| S71 | "tajikistan" OR (MH "USSR+") | (7,376) |
| S70 | (MH "Syria") | (838) |
| S69 | "south sudan" | (224) |
| S68 | "somalia" | (928) |
| S67 | (MH "Rwanda") | (893) |
| S66 | (MH "Niger") | (270) |
| S65 | (MH "Nepal") | (2,571) |
| S64 | (MH "Mozambique") | (719) |
| S63 | (MH "Mali") | (463) |
| S62 | "malawi" | (2,810) |
| S61 | (MH "Liberia") | (462) |
| S60 | (MH "North Korea") OR "north korea" OR (MH "Korea") | (7,734) |
| S59 | north korea | (240) |
| S58 | "guinea bissau" | (192) |
| S57 | (MH "Guinea") OR "guinea" | (3,631) |
| S56 | (MH "Gambia") | (473) |
| S55 | (MH "Ethiopia") OR "ethiopia" | (4,759) |
| S54 | (MH "Eritrea") | (110) |
| S53 | (MH "Democratic Republic of the Congo") OR "congo" | (1,926) |
| S52 | "comoros" | (33) |
| S51 | (MH "Chad") | (130) |
| S50 | (MH "Central African Republic") OR "central african republic" | (161) |
| S49 | (MH "Burundi") or "burundi" | (180) |
| S48 | (MH "Benin") OR "benin" | (588) |
| S47 | (MH "Afghanistan") OR "afghanistan" | (3,765) |
| S46 | S44 AND S45 | (1,055) |
| S45 | S22 OR S23 OR S24 OR S25 OR S26 OR S27 OR S28 OR S29 OR S30 OR S31 OR S32 OR S33 OR S34 OR S35 OR S36 OR S37 OR S38 OR S39 OR S40 | (1,842,060) |
| S44 | S15 OR S16 OR S43 | (3,331) |
| S43 | S41 AND S42 | (2,411) |
| S42 | S13 OR S14 OR S17 OR S18 OR S19 OR S20 OR S21 | (42,039) |
| S41 | S1 OR S2 OR S3 OR S4 OR S5 OR S6 OR S7 OR S8 OR S9 OR S10 OR S11 OR S12 | (10,600) |
| S40 | general pract* | (31,407) |
| S39 | doctor* | (55,325) |
| S38 | physician* | (222,993) |
| S37 | clinician* | (90,895) |
| S36 | (MH "Novice Clinicians+") OR (MH "Clinical Nurse Specialists") OR (MH "Primary Health Care") | (66,260) |
| S35 | (MH "Physicians, Family") | (18,120) |
| S34 | (MH "Family Practice") OR (MH "Practical Nurses") | (28,394) |
| S33 | (MH "Physician Assistant Attitudes") OR (MH "Students, Physician Assistant") OR (MH "Physicians, Emergency") OR (MH "Physician Assistants") | (8,634) |
| S32 | pharmac* | (288,294) |
| S31 | midwi* | (53,914) |
| S30 | nurs* | (802,835) |
| S29 | (MH "Midwife Attitudes") OR (MH "Lay Midwives") OR (MH "Nurse Midwives") OR (MH "Midwives+") OR (MH "Midwifery Service") | (15,647) |
| S28 | (MH "Practical Nurses") OR (MH "Emergency Nurse Practitioners") OR (MH "Nurse Administrators+") OR (MH "Nurse-Managed Centers") OR (MH "Nurse Counselors") OR (MH "Nurse Attitudes") OR (MH "OB-GYN Nurse Practitioners") | (49,808) |
| S27 | (MH "Pharmacist Attitudes") OR (MH "Pharmacists") OR (MH "Pharmacy Technicians") | (14,701) |
| S26 | mid* provid* | (3,366) |
| S25 | health* worker* | (26,968) |
| S24 | (MH "Rural Health Personnel") OR (MH "Community Health Workers") OR (MH "Health Personnel+") | (510,961) |
| S23 | (MH "Physician Assistant Attitudes") OR (MH "Students, Physician Assistant") OR (MH "Physician Assistants") OR (MH "Home Health Aides") OR (MH "Nursing Assistants") OR (MH "Medical Assistants") | (14,830) |
| S22 | (MH "Community Health Workers") OR (MH "Rural Health Personnel") OR (MH "Community Health Centers+") OR (MH "Community Health Nursing+") OR (MH "Hospitals, Community") OR (MH "Health Personnel+") OR (MH "Community Health Services+") OR (MH "Community Medicine") OR (MH "Community Networks") OR (MH "Community Practitioners' and Health Visitors' Association") | (862,385) |
| S21 | menstrua* regulat* | (116) |
| S20 | termina* N2 fetus | (77) |
| S19 | termina* N2 baby | (11) |
| S18 | termin* pregnan* | (3,079) |
| S17 | (MH "Pregnancy Reduction, Multifetal") OR (MH "Pregnancy, Unplanned") OR (MH "Pregnancy Outcomes") | (23,878) |
| S16 | medic* abort* | (1,364) |
| S15 | surgi* abort* | (447) |
| S14 | abort* | (19,052) |
| S13 | (MH "Abortion, Induced+") OR "induced abortion" | (9,489) |
| S12 | "dilation and extraction" | (9) |
| S11 | (MH "Cervix Dilatation and Effacement") OR "dilation and evacuation" | (920) |
| S10 | (MH "Dilatation and Curettage+") OR (MH "Curettage+") OR (MH "Dilatation+") OR (MH "Vacuum Curettage") OR "dilation and curettage" | (5,317) |
| S9 | eva | (608) |
| S8 | (MH "Vacuum Curettage") OR (MH "Vacuum Extraction, Obstetrical") OR "electric vacuum aspiration" | (743) |
| S7 | "mva" | (552) |
| S6 | (MH "Vacuum Curettage") OR (MH "Vacuum Extraction, Obstetrical") OR "manual vacuum aspiration" | (794) |
| S5 | (MH "Abortifacient Agents+") OR "abortifacient" | (2,630) |
| S4 | (MH "Mifepristone") OR "mifepristone" | (1,173) |
| S3 | mifepristone | (1,174) |
| S2 | (MH "Misoprostol") OR "misoprostol" | (2,061) |
| S1 | misoprostol | (2,061) |

**Appendix S2: PRISMA Checklist**

| **Section/topic** | **#** | **Checklist item** | **Reported on page #** |
| --- | --- | --- | --- |
| **TITLE** | | |  |
| Title | 1 | Identify the report as a systematic review, meta-analysis, or both. | 2 |
| **ABSTRACT** | | |  |
| Structured summary | 2 | Provide a structured summary including, as applicable: background; objectives; data sources; study eligibility criteria, participants, and interventions; study appraisal and synthesis methods; results; limitations; conclusions and implications of key findings; systematic review registration number. | 2 |
| **INTRODUCTION** | | |  |
| Rationale | 3 | Describe the rationale for the review in the context of what is already known. | 4 |
| Objectives | 4 | Provide an explicit statement of questions being addressed with reference to participants, interventions, comparisons, outcomes, and study design (PICOS). | 6 |
| **METHODS** | | |  |
| Protocol and registration | 5 | Indicate if a review protocol exists, if and where it can be accessed (e.g., Web address), and, if available, provide registration information including registration number. | NA |
| Eligibility criteria | 6 | Specify study characteristics (e.g., PICOS, length of follow-up) and report characteristics (e.g., years considered, language, publication status) used as criteria for eligibility, giving rationale. | 6 |
| Information sources | 7 | Describe all information sources (e.g., databases with dates of coverage, contact with study authors to identify additional studies) in the search and date last searched. | 6 |
| Search | 8 | Present full electronic search strategy for at least one database, including any limits used, such that it could be repeated. | Appendix S1.1-1.6 |
| Study selection | 9 | State the process for selecting studies (i.e., screening, eligibility, included in systematic review, and, if applicable, included in the meta-analysis). | 6 |
| Data collection process | 10 | Describe method of data extraction from reports (e.g., piloted forms, independently, in duplicate) and any processes for obtaining and confirming data from investigators. | 7 |
| Data items | 11 | List and define all variables for which data were sought (e.g., PICOS, funding sources) and any assumptions and simplifications made. | 6/7 |
| Risk of bias in individual studies | 12 | Describe methods used for assessing risk of bias of individual studies (including specification of whether this was done at the study or outcome level), and how this information is to be used in any data synthesis. | 6 |
| Summary measures | 13 | State the principal summary measures (e.g., risk ratio, difference in means). | na |
| Synthesis of results | 14 | Describe the methods of handling data and combining results of studies, if done, including measures of consistency (e.g., I^2^) for each meta-analysis. | 6 |

**Appendix S3: PI(C)OS Search Terms and Inclusion Criteria**

| **PI(C)OS** | | **Search terms** | **Inclusion Criteria** |
| --- | --- | --- | --- |
| Population | Providers | Nurse  Midwife  Community health worker  Mid-level provider/practitioner  General/Family Physician/practitioner  Obstetrics and Gynaecologist  Pharmacist  Healthcare worker | Abortion services provided in primary care settings  Private and public healthcare services |
|  | Countries | **Low income countries:** Afghanistan, Benin, Burkina Faso, Burundi, Central African Republic, Chad, Comoros, Congo Democratic Republic, Eritrea, Ethiopia, Gambia ,Guinea, Guinea Bissau, North Korea, Democratic People Republic of Korea, Liberia, Malawi, Mali, Mozambique, Nepal, Niger, Rwanda, Somalia, South Sudan, Syria, Tajikistan, Tanzania, Togo, Uganda, Yemen Republic, Zimbabwe  **Lower-middle income countries:** Angola, Bangladesh, Bhutan, Bolivia, Cape Verde, Cambodia, Cameroon, Cote d’Ivoire, Ivory Coast, Djibouti, Georgia, Ghana, India, Indonesia, Kenya, Kiribati, Kosovo, Kyrgyz Republic, Kyrgyzstan, Lesotho, Micronesia, Moldova, Mongolia, Morocco, Myanmar, Nigeria, Pakistan, Papua New Guinea, Sao Tome and Principe, Solomon Islands, Sri Lanka, Sudan, Swaziland, Eswatini, Timor Lester, Tunisia, Ukraine, Uzbekistan, Vanuatu, Vietnam, Zambia  **Upper-middle income countries:** Albania, Algeria, American Samoa, Armenia, Azerbaijan, Belarus, Belize, Bosna and Herzegovina, Botswana, Brazil, Bulgaria, China, Colombia, Costa Rica, Cuba, Dominica, Equatorial Guinea, Ecuador, Fiji, Grenada, Guatemala, Guyana, Iran, Jordan, Kazakhstan, Lebanon, Libya, Macedonia, Malaysia, Maldives, Mauritius, Mexico, Montenegro, Namibia, Nauru, Paraguay, Peru, Romania, Russia, Russian Federation, Samoa, Serbia, South Africa, St Lucia, St Vincent and the Grenadines, Thailand, Turkey, Turkmenistan, Tuvalu, Venezuela | |
| Intervention | Procedure | Manual vacuum aspiration/MVA  Electric vacuum aspiration/EVA  Misoprostol  Mifepristone  Dilatation and curettage/D&C  Dilatation and evacuation/D&E | Early or first trimester abortions (Under 12 weeks)  Comprehensive abortion care  Safe abortion care |
|  | Variations of “abortion” | Induced abortion  Termination of pregnancy/baby/foetus  Menstrual regulation  Surgical abortion  Medical abortion^[[1]](#footnote-1)^ |  |
| Outcome | NA | NA | Addressing any of the following themes:   - Medical care facility - Medication and equipment used - Provider competencies and attitudes - Post-abortion counselling - Abortion morbidity and mortality rates - Client satisfaction |
| Study Design | NA | NA | Only peer reviewed, primary studies |

**Appendix S4: Detailed list of included papers**

| **First author** | **Year published [studied]** | **Country** | **Abortion legality^1^** | **Provider** | **Setting Type** | **Traini-ng?** | **Sample size** | **Study type (Description)** | **Quality^[[2]](#footnote-2)^** | **Study limitations** |
| --- | --- | --- | --- | --- | --- | --- | --- | --- | --- | --- |
| Andersen et al. [26] | 2016 [2010-2014] | Nepal | 4 | ANM^2^ | PCF^4^ | Y | 25187 | **Descriptive program evaluation** (Site assessments, and quantitative data collected on service provision and quality) | Medium | No baseline observations; unmatched data |
| Assefa et al. [27] | 2019  [2015] | Ethiopia | 4 | Nurse, Health Officers, Midwives | Health Centres | N | 405 | **Cross-sectional study** (Structured questionnaires for MLPs) | High | Did not investigate structure themes, no private clinics |
| Banerjee et al. [28] | 2010 [2005-2007] | India | 3 | Doctors (Majority OB/GYN^3^) | Small clinical set-up | Y | 60 | **Descriptive program evaluation** (Semi-structured questionnaire at baseline and follow-up, and in-depth interview with doctors) | Medium | Unmatched data, no comparison group, no MLP studied |
| Benson et al. [29] | 2017 [2012-2015] | India, Nepal, Nigeria | 3, 4, 1 | Physicians, ANM^2^, midwives | PCF^4^ | Y | 3435 | **Prospective cohort study** (Questionnaires for providers at baseline and follow-up over a 4-year period) | Medium | No comparison group; poor quality of baseline data |
| Johnson et al. [30] | 2018 [2014-2015] | Kyrgyzstan | 4 | Midwives and family nurses | Referral centers and FOPs^5^ | Y | 554 | **Implementation study** (Abortion outcomes of clients, and questionnaires for clients at exit phase) | Medium | Conditions in study may not apply to actual provision |
| Kawonga et al. [31] | 2008 [2004] | South Africa | 4 | Did not specify | PCF^4^ | Y | 290 | **Operation study** (Abortion outcomes of clients and exit interview with clients. Qualitative interviews with 5 providers) | Low | Interview results are not representative of population |
| KC NP et al. [32] | 2011 [2011] | Nepal | 4 | ANM^2^ and senior nurses | PHC^6^ and HP^7^ | Y | 1799 | **Implementation study** (Abortion outcomes of clients and interview on client's experience; and qualitative site evaluation) | Medium | Incomplete baseline data |
| Marlow et al. [33] | 2016 [2013-2014] | Bangladesh | 1 | Did not specify | PCF^4^ and Pharmacy | NA | 10 | **Qualitative study** (in-depth interview to understand the perspectives women's experience on medical abortion) | High | Likely influenced by individual biasedness |
| Mundle et al. [34] | 2007 [2004-2005] | India | 3 | Doctors | PHC^6^ | Y | 150 | **Implementation study** (Abortion outcome of clients, questionnaires on client experience and daily symptom diary cards for clients) | High | May not apply to other PHCs in India |
| Okonofua et al. [35] | 2011 [2009] | Nigeria | 1 | Doctors (Mostly GP^8^ and OB/GYN^3^) | Private clinics | N | 122 | **Cross-sectional study** (Structured questionnaires for doctors) | High | Possible under-reporting due to legal issues |
| Okonofua et al. [36] | 2005 [2000] | Nigeria | 1 | Doctors (Majority GP^8^ and OB/GYN^3^) | Private clinics | N | 323 | **Cross-sectional study** (Structured questionnaires for doctors) | High | Possible under-reporting due to legal issues |
| Puri et al. [37] | 2018 [2014-2015] | Nepal | 4 | ANM^2^ | PCF^4^ and Pharmacy | Y | 605 | **Prospective cohort study (**Structured questionnaire for clients comparing the services in pharmacy vs. public health facility) | Medium | Confounding factor, small sample size, not representative of population |
| Puri et al. [38] | 2014 [2011-2012] | Nepal | 4 | ANM^2^ | PHC^6^ and HP^7^ | Y | 241 | **Implementation study** (Exit interviews with client, Semi-structured interviews with ANMs) | Low | May not apply to other PCFs in Nepal |
| Ramachandar and Pelto [39] | 2005 [2005] | India | 3 | Doctors, nurses, midwives, and pharmacists | PCF^4^ and Pharmacy | N | 40 | **Qualitative study** (in-depth interview with providers on medical abortion) | Medium | Likely influenced by individual biasedness |
| Rocca et al. [40] | 2018 [2014-2015] | Nepal | 4 | ANM^2^ | PCF^4^ and Pharmacy | Y | 605 | **Prospective cohort study** (Structured questionnaire for clients comparing the services in pharmacy vs. public health facility) | High | Confounding factor, small sample size, not representative of population |
| Tamang et al. [41] | 2017 [2009-2010] | Nepal | 4 | ANM^2^ + staff nurses (MLP) vs. doctors | District hospital^10^ | Y | 1077 | **Randomised controlled trial** (Exit survey "Acceptability form" administered to ascertain women's experience) | High | Measures of satisfaction may be subjective, and affected by confounding factors |
| Tran et al. [42] | 2010 [2007-2008] | DPRK | 4 | Doctors | RHC^9^ | Y | 199 | **Implementation study** (Abortion outcomes of clients and exit survey with clients) | High | Conditions in study may not apply to actual provision |
| Warriner et al. [43] | 2011 [2009-2010] | Nepal | 4 | ANM^2^ + staff nurses (MLP) vs. doctors | District hospital^10^ | Y | 1077 | **Randomised controlled trial** (Client abortion outcomes) | High | May not apply to other PCF in Nepal; possible convergence of outcomes |

1. Abortion legality: (1) To save woman's life; (2) To save life. preserve physical and mental health; (3) To save life. preserve physical and mental health, and on socioeconomic grounds; (4) On request; 2. ANM = Auxiliary nurse-midwives; 3. OB/GYN = Obstetrician and Gynaecologists; 4. PCF = Primary care facility; 5. FOP = Felsher Obstetric Points; 6. PHC = Primary health centres; 7. HP = Health posts; 8. GP = General practitioner; 9. RHC = Reproductive health clinics; 10. District hospital is considered as part of its primary care in Nepal.

**Appendix S5: Exclusion List**

| **#** | **Authors** | **Title** | **Study Design** | **Region, Country** | **Date of Study** | **Reason for Exclusion** |
| --- | --- | --- | --- | --- | --- | --- |
| 1 | Alam A., Reichenbach L., Huda F.A., Ahmed A. and Ngo T.D. | Pharmacy and Medicine Sellers' Knowledge and provision of misoprostol for menstrual regulation in Bangladesh | Cross-sectional study | Bangladesh | 2012 | Not primary study |
| 2 | Acharya R., Kalyanwala S. | Knowledge, attitudes, and practices of certified providers of medical abortion: evidence from Bihar and Maharashtra, India. | Cross-sectional study | India | 2012 | Not disaggregated data |
| 3 | Begum F., Zaidi S., Fatima P., Shamsuddin L., Anowar-ul-Azim A.K.M., Begum R.A. | Improving manual vacuum aspiration service delivery, introducing misoprostol for cases of incomplete abortion, and strengthening postabortion contraception in Bangladesh. | Cross-sectional study | Bangladesh | 2014 | Not primary care setting |
| 4 | Billings DL, Walker D, Paso GMD, Clark KA, Dayananda I. | Pharmacy worker practices related to use of misoprostol for abortion in one Mexican state. | Cross-sectional study | Mexico | 2009 | Pharmacy Setting |
| 5 | Bridgman-Packer D., Kidanemariam S. | The implementation of safe abortion services in Ethiopia | Case-series; qualitative | Ethiopia | 2018 | Wrong study aim |
| 6 | Chavkin W., Baffoe P., Awoonor-Williams K. | Implementing safe abortion in Ghana: "We must tell our story and tell it well". | Case Study | Ghana | 2018 | Wrong study aim |
| 7 | Ciren B., Fjeld H. | Pragmatics of everyday life: A qualitative study of induced abortion among Tibetan women in Lhasa | Qualitative study | Tibet | 2019 | Not primary care setting |
| 8 | Diamond-Smith N, Percher J, Saxena M, Dwivedi P, Srivastava A. | Knowledge, provision of information and barriers to high quality medication abortion provision by pharmacists in Uttar Pradesh, India. | Cross-sectional study | India | 2019 | Pharmacy Setting |
| 9 | Dickinson-Tetteh K. and Billings D.L. | Abortion care services provided by registered midwives in South Africa | Prospective cohort study | South Africa | 2002 | Not disaggregated data |
| 10 | Fetters T, Raisanen K, Mupeta S, Malisikwanda I, Vwalika B, Osur J | Using a harm reduction lens to examine post-intervention results of medical abortion training among Zambian pharmacists. | Prospective cohort study | Zambia | 2014 | Pharmacy Setting |
| 11 | Foster A.M., Arnott G. and Hobstetter M. | Community-based distribution of misoprostol for early abortion: evaluation of a program along the Thailand-Burma border | Prospective cohort study | Thailand-Burma border | 2017 | Not primary care setting |
| 12 | Gallo M.F. Gebreselassie H., Victorino M.T.A., Dgedge M., Jamisse L. and Bique C | An assessemnt of abortion services in public health facilities in Mozambique: Women's and Provider's perspectives | Cross-sectional study | Mozambique | 2004 | Not disaggregated data |
| 13 | Ganatra B, Manning V, Pallipamulla SP. | Availability of Medical Abortion Pills and the Role of Chemists: A Study from Bihar and Jharkhand, India. | Cross-sectional study | India | 2005 | Pharmacy Setting |
| 14 | Ganatra B., Bygdeman M., Phan B.T., Nguyan D.V. and Vu M. L. | From research to reality: the challenges of introducing medical abortion into service delivery for Vietnam | Cross-sectional study | Vietnam | 2004 | Not primary care setting |
| 15 | Ganle JK, Busia NT, Maya E. | Availability and prescription of misoprostol for medical abortion in community pharmacies and associated factors in Accra, Ghana. | Cross-sectional study | Ghana | 2018 | Pharmacy Setting |
| 16 | Gupta P., Iyengar S.D., Ganatra B., Johnston H.B. and Iyengar K. | Can community health workers play a greater role in increasing access to medical abortion services? A qualitative study | Cross-sectional study | Rajasthan, India | 2017 | Wrong study aim |
| 17 | Harries J., Constant D. | Providing safe abortion services: Experiences and perspectives of providers in South Africa. | Secondary Study | South Africa | 2020 | Not primary study |
| 18 | Hendrickson C, Fetters T, Mupeta S, Vwallika B, Djemo P, Raisanen K. | Client-pharmacy worker interactions regarding medical abortion in Zambia in 2009 and 2011. | Cross-sectional study | Zambia | 2015 | Pharmacy Setting |
| 19 | Huda FA, Mahmood HR, Alam A, Ahmmed F, Karim F, Sarker BK, et al. | Provision of menstrual regulation with medication among pharmacies in three municipal districts of Bangladesh: a situation analysis. | Cross-sectional study | Bangladesh | 2018 | Pharmacy Setting |
| 20 | Huda FA, Ngo TD, Ahmed A, Alam A, Reichenbach L. | Availability and provision of misoprostol and other medicines for menstrual regulation among pharmacies in Bangladesh via mystery client survey. | Cross-sectional study | Bangladesh | 2013 | Pharmacy Setting |
| 21 | IPAS | Medical Abortion in Bihar and Jharkhand: A study of service providers, chemists, women and men | Cross-sectional study | ndia | 2005 | Not primary study |
| 22 | Iyengar K. and Iyengar S.D. | Improving access to safe abortion in a rural primary care setting in India: experience of a service delivery intervention | Cross-sectional study | India | 2016 | Not primary care setting |
| 23 | Jejeebhoy S.J., Kalyanwala S., Mundle S., Tank J., Zavier A.J.F., Kumar R., Acharya R. and Jha N. | Feasibility of expanding the medication abortion provider base in India to include Ayurvedic physicians and nurses | RCT (Equivalence study) | India | 2012 | Not primary care setting |
| 24 | Jejeebhoy S.J., Kalyanwala S., Zavier A.J.F., Kumar R., Mundle S., Tank J., Acharya R. and Jha N. | Can nurses perform manual vacuum aspiration (MVA) as safely and effectively as physicians? Evidence from India | RCT (Equivalence study) | India | 2011 | Not primary care setting |
| 25 | Kapp N., Methazia J., Eckersberger E., Griffin R., Bessenaar T. | Label comprehension of a combined mifepristone and misoprostol product for medical abortion: A pilot study in South Africa. | Cross-sectional study | South Africa | 2020 | Not primary care setting |
| 26 | Karki C., Ojha M. and Rayamajhi R.T. | Baseline survey on functioning of abortion services in government approved CAC centres in three pilot districts of Nepal | Cross-sectional study | Nepal | 2012 | Wrong study aim |
| 27 | Kroeger A., Ochoa H., Arana B., Diaz A., Rizzo N., and Flores W. | Inadequate drug advice in the pharmacies of Guatemala and Mexico: the scale of the problem and explanatory factors | Cross-sectional study | Chiapas, Mexico and Guatemala | 2001 | Wrong study aim |
| 28 | Lara D, Abuabara K, Grossman D, Díaz-Olavarrieta C. | Pharmacy provision of medical abortifacients in a Latin American city. | Cross-sectional study | a Latin American city | 2006 | Pharmacy Setting |
| 29 | Lara D, García SG, Wilson KS, Paz F. | How Often and Under Which Circumstances Do Mexican Pharmacy Vendors Recommend Misoprostol To Induce an Abortion? | Cross-sectional study | Mexico | 2011 | Pharmacy Setting |
| 30 | Moller A., Ofverstedt S. and Siwe K. | Proud, not yet satisfied: the experiences of abortion service providers in the Kathmandu Valley, Nepal | Cross-sectional study | Nepal | 2012 | Wrong study aim |
| 31 | Ngo T.D., Free C., Le H.T., Edwards P., Pham K.H.T., Nguyen Y.B.T. and Nguyen T.H. | Attributes and prospectives of public providers related to provision of medical abortion at public health facilities in Vietnam: a cross sectional study in three provinces | Cross-sectional study | Hanoi, Khanh Hoa and Ho Chi Minh, Vietnam | 2014 | Wrong study aim |
| 32 | Ngo T.D., Park M.H. and Nguyen T.H. | Pharmacy workers' knowledge and provision of abortifacients in Ho Chi Minh City, Vietnam | Cross-sectional study | Ho Chi Minh, Vietnam | 2011 | Not primary study |
| 33 | Powell-Jackson T, Acharya R, Filippi V, Ronsmans C. | Delivering Medical Abortion at Scale: A Study of the Retail Market for Medical Abortion in Madhya Pradesh, India. | Cross-sectional study | India | 2015 | Pharmacy Setting |
| 34 | Ramachandar L. and Pelto R.J. | Abortion providers and safety of abortion: a community-based study in a rural district of Tamil Nadu, India | Cross-sectional study (qualitative) | India | 2004 | Not disaggregated data |
| 35 | Reiss K, Footman K, Akora V, Liambila W, Ngo TD. | Pharmacy workers’ knowledge and provision of medication for termination of pregnancy in Kenya. | Cross-sectional study | Kenya | 2016 | Pharmacy Setting |
| 36 | Rocca C., Puri M., Blum M., Shrestha P., Grossman D. | Expanding access to medication abortion through nurses in pharmacies in Nepal: Safety and effectiveness | Prospective cohort study | Nepal | 2015 | No full paper |
| 37 | Rocca C., Puri M., Blum M., Shrestha P., Grossman D., Harper C. | Safety and effectiveness of access to medication abortion from pharmacy clinics in Nepal | Cross-sectional study | Nepal | 2015 | No full paper |
| 38 | Samari, Puri, Cohen, Blum, Rocca. | Pharmacy Provision of Medication Abortion in Nepal: Pharmacy Owner and Worker Perspectives. | Qualitative study | Nepal | 2018 | Pharmacy Setting |
| 39 | Sayami J.T. | Trends in Comprehensive Abortion Care (CAC) and characteristics of women receiving abortion care in a tertiary hospital in Nepal | Cross-sectional study | Nepal | 2019 | Not primary care setting |
| 40 | Sharma R., Radhakrishnan G., Mehdiratta A., and Gupta R. | Awareness, attitude, and acceptability for abortion law among MTP seekers at a tertiary care center of east Delhi | Cross-sectional study | India | 2019 | Not primary care setting |
| 41 | Sri B.S. and Ravindran T.K.S. | Medical abortion: understanding the perspectives of rural and marginalised women from rural South India | Cross-sectional study | India | 2012 | Wrong study aim |
| 42 | Sundaram A., Juarez F., Ahiadeke C., Bankole A. and Blades N. | The impact of Ghana's R3M programme on the provision of safe abortions and postabortion care | Prospective cohort study | Ghana | 2015 | Not primary care setting |
| 43 | Tamang A, Puri M, Lama K, Shrestha P. | Pharmacy workers in Nepal can provide the correct information about using mifepristone and misoprostol to women seeking medication to induce abortion. | Non-randomised study | Nepal | 2014 | Pharmacy Setting |
| 44 | Tamang A, Puri M, Masud S, Karki DK, Khadka D, Singh M, et al. | Medical abortion can be provided safely and effectively by pharmacy workers trained within a harm reduction framework: Nepal. | Non-randomised study | Nepal | 2018 | Pharmacy Setting |
| 45 | Warriner I.K., Meirik O., Hoffman M., Morroni C., Harries J., My Huong N.T., Vy N.D. and Seuc A.H. | Rates of complications in first trimester manual vacuum aspiration abortion done by doctors and mid-level providers in South Africa and Vietnam: a randomised controlled equivalence trial | RCT (Equivalence study) | South Africa and Vietnam | 2006 | Not primary care setting |
| 46 | Zavier, A J Francis; Santhya, K G; Jejeebhoy, Shireen J | Abortion among married young women: findings from a community-based study in Rajasthan and Uttar Pradesh, India. | Cross-sectional study | India | 2019 | Wrong study aim |

**Appendix S6: Countries sorted on abortion law and income group**

**Appendix S7: Mixed Method Appraisal Tool (Quality Assessment)**

|  |  |  | **1. QUALITATIVE STUDIES** | | | | | **COMMENTS** |
| --- | --- | --- | --- | --- | --- | --- | --- | --- |
| Author | Year | Reference | 1.1. Is the qualitative approach appropriate to answer the research question? | 1.2. Are the qualitative data collection methods adequate to address the research question? | 1.3. Are the findings adequately derived from the data? | 1.4. Is the interpretation of results sufficiently substantiated by data? | 1.5. Is there coherence between qualitative data sources, collection, analysis and interpretation? |  |
| Marlow | 2015 | Marlow HM, Biswas K, Griffin R, Menzel J. Women’s experiences with medication for menstrual regulation in Bangladesh. Culture, health & sexuality. 2016 Mar 3;18(3):349-60. | YES. The research question seeks for women's opinions and experiences. | YES. In-depth interviews were conducted in Bangla, lasted for 1-2 hours and were tape recorded, transcribed and translated. English transcripts were coded and themes were derived. | YES. Inductive and deductive methods were used for qualitative description. | YES. There were appropriate usage of quotes to substantiate interpretation of results | YES. Data collection, analysis and interpretation was coherent. | 100% HIGH QUALITY Did not mention any study limitations |
| Ramachan  dar | 2005 | Ramachandar L, Pelto PJ. Medical Abortion in Rural Tamil Nadu, South India: A Quiet Transformation. Reproductive Health Matters. [Online] 2005;13(26): 54–64. Available from: doi:10.1016/s0968-8080(05)26195-5 | NO. Mixed methods can be a better approach for this research question as there are some sections describing quantitative data, but not presented in quantitative format. | YES. In-depth, open-ended interviews were conducted, and interviewees were selected using a non-random, snowball sampling technique to ensure representation . But no coding of qualitative data and organising into thematic segments were mentioned in the methods. | CAN'T TELL. It was unclear how qualitative data was analysed. | YES. All interpretentions in results were substantiated by quotes or frequency of occurrence. | NO. There was little explanation on qualitative data analysis segment. | 40% MEDIUM QUALITY  No limitations mentioned in study |

|  |  |  | **2. RANDOMIZED CONTROLLED TRIALS** | | | | | **COMMENTS** |
| --- | --- | --- | --- | --- | --- | --- | --- | --- |
| Author | Year | Reference | 2.1. Is randomization appropriately performed? | 2.2. Are the groups comparable at baseline? | 2.3. Are there complete outcome data? | 2.4. Are outcome assessors blinded to the intervention provided? | 2.5 Did the participants adhere to the assigned intervention? |  |
|  |  |  |  |  |  |  |  |  |
| Tamang | 2017 | Tamang A, Shah IH, Shrestha P, Warriner IK, Wang D, Thapa K, et al. Comparative satisfaction of receiving medical abortion service from nurses and auxiliary nurse-midwives or doctors in Nepal: results of a randomized trial. Reproductive Health. [Online] 2017;14(1). Available from: doi:10.1186/s12978-017-0438-7 | YES. Eligible women were randomly assigned either groups using a computer-generated randomization scheme classified by the participating center with a block size of six. Researchers and participants were unaware of assignment - the random allocation was indicated in sealed opaque envelopes, numbered consecutively | YES. Baseline measurements were not relevant in this study. Baseline characteristics of women in both groups were collected and they were comparable. | YES. 4% loss to follow-up in both groups during study. | NO. It was not possible for participant to be completely blinded to intervention. | YES. All participants adhered to assigned intervention. | 80% HIGH QUALITY |
| Warriner | 2011 | Warriner I, Wang D, Huong NM, Thapa K, Tamang A, Shah I, et al. Can midlevel health-care providers administer early medical abortion as safely and effectively as doctors? A randomised controlled equivalence trial in Nepal. The Lancet. [Online] 2011;377(9772): 1155–1161. Available from: doi:10.1016/s0140-6736(10)62229-5 | YES. Eligible women were randomly assigned either groups using a computer-generated randomization scheme classified by the participating center with a block size of six. Researchers and participants were unaware of assignment - the random allocation was indicated in sealed opaque envelopes, numbered consecutively | YES. Baseline measurements were not relevant in this study. Baseline characteristics of women in both groups were collected and they were comparable. | YES. 4% loss to follow-up in both groups during study. | NO. It was not possible for participant to be completely blinded to intervention. | YES. All participants adhered to assigned intervention. | 80% HIGH QUALITY |

|  |  |  | **3. NON-RANDOMIZED STUDIES** | | | | | **COMMENTS** |
| --- | --- | --- | --- | --- | --- | --- | --- | --- |
| Author | Year | Reference | 3.1. Are the participants representative of the target population? | 3.2. Are measurements appropriate regarding both the outcome and intervention (or exposure)? | 3.3. Are there complete outcome data? | 3.4. Are the confounders accounted for in the design and analysis? | 3.5. During the study period, is the intervention administered (or exposure occurred) as intended? |  |
| Andersen | 2016 | Andersen KL, Basnett I, Shrestha DR, Shrestha MK, Shah M, Aryal S. Expansion of Safe Abortion Services in Nepal Through Auxiliary Nurse-Midwife Provision of Medical Abortion, 2011-2013. Journal of Midwifery &amp; Women's Health. [Online] 2016;61(2): 177–184. Available from: doi:10.1111/jmwh.12419 | CAN'T TELL. There was little information on the target population, and no exclusion or inclusion criteria for choosing providers or participants were mentioned in the study. But facilities used had a selection criteria. | NO. Variables are clearly defined. Standardised clinical skills assessment checklist was used. But figures could be better utilised to present data. There was also little baseline observations. | YES. There was complete outcome data. | NO. There was no matching of pre-and-post intervention data. In addition, the each year focused on a different set of providers to evaluate the outcome of scaling up this intervention | CAN'T TELL. Intervention was administered as intended. But there was no mention of any co-exposure that may have happened at the same time. | 20% LOW QUALITY |
| Benson | 2017 | Benson J, Healy J, Dijkerman S, Andersen K. Improving health worker performance of abortion services: an assessment of post-training support to providers in India, Nepal and Nigeria. Reproductive Health. [Online] 2017;14(1). Available from: doi:10.1186/s12978-017-0416-0 | YES. | YES | YES | NO | CAN'T TELL | 60% MEDIUM QUALITY |
| Puri | 2015 | Puri M, Tamang A, Shrestha P, Joshi D. The role of auxiliary nurse-midwives and community health volunteers in expanding access to medical abortion in rural Nepal. Reproductive Health Matters. [Online] 2014;22(sup44): 94–103. Available from: doi:10.1016/s0968-8080(14)43784-4 | CAN'T TELL. There was little information on the target population, and no exclusion or inclusion criteria for choosing providers or participants were mentioned in the study. | NO. Variables are clearly defined. Accuracy was marked down as an error was spotted in table 5. No mention of validity or reliability of questionaires, semi-structured interview questions. | CAN'T TELL. Did not state no-response rate | YES. Pre- and post- intervention data were matched. | CAN'T TELL. Intervention was administered as indended. But there was no mention of any co-exposure that may have happened at the same time even though data may hint towards this (differences in comparison group was not acknowledged and explained). | 20% LOW QUALITY. |
| Puri | 2018 | Puri MC, Harper CC, Maharjan D, Blum M, Rocca CH. Pharmacy access to medical abortion from trained providers and post-abortion contraception in Nepal. International Journal of Gynecology &amp; Obstetrics. [Online] 2018;143(2): 211–216. Available from: doi:10.1002/ijgo.12595 | CAN'T TELL. There was little information on the target population, but criterias were mentioned for exclusion of participants in the study. | NO. Variables are clearly defined, and only parts of measurements were justified. There was no mention of pilot stage to assess feasibility and test instruments. | YES. < 1% loss to follow-up. | YES. Data was stratified and regression was performed to take into account of confounding variables. | YES. Intervention took place as planned. No contaminations and co-interventions were present. | 60% MEDIUM QUALITY |
| Rocca | 2018 | Rocca CH, Puri M, Shrestha P, Blum M, Maharjan D, Grossman D, et al. Effectiveness and safety of early medication abortion provided in pharmacies by auxiliary nurse-midwives: A non-inferiority study in Nepal. Plos One. [Online] 2018;13(1). Available from: doi:10.1371/journal.pone.0191174 | YES. There was clear description of target population, and women who did not meet eligibility criteria were excluded from study - reasons for exclusion were also included in the study. | YES. Variables were clearly defined. A pilot test was conducted to assess feasibility and test instruments. | YES. < 1% loss to follow-up. | YES. Data was stratified and regression was performed to take into account of confounding variables. | YES. Intervention took place as planned. No contaminations and co-interventions were present. | 100% HIGH QUALITY |

|  |  |  | **4. QUANTITATIVE DESCRIPTIVE STUDIES** | | | | | **COMMENTS** |
| --- | --- | --- | --- | --- | --- | --- | --- | --- |
| Author | Year | Reference | 4.1. Is the sampling strategy relevant to address the research question? | 4.2. Is the sample representative of the target population? | 4.3. Are the measurements appropriate? | 4.4. Is the risk of nonresponse bias low? | 4.5. Is the statistical analysis appropriate to answer the research question? |  |
| Assefa | 2019 | Assefa E.M. Knowledge, attitude and practice (KAP) of health providers towards safe abortion provision in Addis Ababa health centers. BMC Women’s Health. [Online] 2019; 19: 138. Available from: https://doi.org/10.1186/s12905-019-0835-x | YES. Probability sampling was used to select health centers and providers. | YES. Clear description of target population and sample. A multi-stage cluster sampling was used to select sample | CAN’T TELL. Variables are clearly defined and accurately measured. Measurements are justified and appropriate to research question. Questionnaires were pre-tested prior to data collection. However, mean score on knowledge and attitude questions was not indicated, and unclear on how authors derived the scoring. | YES. 3.6% did not respond | YES. Normality plots tests (Kolmogorov-Smirnov & Q-Q plot) were used. | 80% HIGH QUALITY  This study did not explain how questions were scored. |
| Johnson | 2018 | Johnson BR, Maksutova E, Boobekova A, Davletova A, Kazakbaeva C, Kondrateva Y, et al. Provision of medical abortion by midlevel healthcare providers in Kyrgyzstan: testing an intervention to expand safe abortion services to underserved rural and periurban areas. Contraception. [Online] 2018;97(2): 160–166. Available from: doi:10.1016/j.contraception.2017.11.002 | YES. Non-probability: Patients who visited the facility at a certain time were chosen for the study. | YES. Clear inclusion and exclusion criteria to choose patients, and explanation was given as to why eligible women were not included in study. Efforts were made to collect missing data. | YES. Variables are clearly defined and accurately measured, the measurements are justified and appropriate to research question. Study did not state not validated or pre-tested prior to data collection. | NO. 40% did not acceptability survey at exit phase | CAN'T TELL. Study did not specify statistical method used, only described data management. | 60% HIGH QUALITY This study did not mention their study limitations at all (implementation study) |
| KC NP | 2011 | KC NP,Basnett I, Sharma SK, Bhusal CL, Parajuli RR, Andersen KL. Increasing Access to Safe Abortion Services Through Auxiliary Nurse Midwives Trained as Skilled Birth Attendants. Kathmandu Univ Med J 2011;36(4):260-66. | YES. Non-probability: Patients who visited the facility at a certain time were chosen for the study. | YES. Clear inclusion and exclusion criteria used to choose facilities. But it was unclear on the patients profiles, or reasons for any patients excluded from their study. | NO. Variables did not have clear definition - did not explain how "provider performance score" was derived. Study also did not justify how these measurements were decided. | YES. 2.2% patients were lost to follow-up. | NO. Basic measurements, frequency and percentages were used, but more sophisticated measurements could offer greater insight. Graphs are unclear, and were not optimised for interpretation | 60% MEDIUM QUALITY This study did not mention their study limitations at all (Implementation study) |
| Mundle | 2007 | Mundle S, Elul B, Anand A, Kalyanwala S, Ughade S. Increasing access to safe abortion services in rural India: experiences with medical abortion in a primary health center. Contraception. [Online] 2007;76(1): 66–70. Available from: doi:10.1016/j.contraception.2007.03.010 | YES. Non-probability: Patients who visited the facility at a certain time were chosen for the study. | YES. Clear inclusion and exclusion criteria to choose patients, and explanation was given as to why eligible women were not included in study. Efforts were made to collect missing data. | YES. Variables are clearly defined and accurately measured. Measurements are justified and appropriate to research questions: they were standardised questtionaires that were used in several medical abortion studies in India. | YES. 3.3% patients were lost to follow-up. | YES. Data were analysed using SPSS and STATA, and presented clearly with appropriate intterpretation | 100% HIGH QUALITY |
| Okonofua | 2011 | Okonofua FE, Hammed A, Abass T, Mairiga AG, Mohammed AB, Adewale A, et al. Private Medical Providers' Knowledge and Practices Concerning Medical Abortion in Nigeria. Studies in Family Planning. [Online] 2011;42(1): 41–50. Available from: doi:10.1111/j.1728-4465.2011.00263.x | YES. Non-probability: Providers at all private medical clinics were chosen for study | YES. All providers in the population were contacted for survey, those that did not provider maternal healthcare were excluded. | YES. Variables had clear definition, structured questionaire was pre-tested and rigorous methods were used to ensure its delivery, and justified. | YES. 10% non-reponse as all these providers did not provide abortion services. | NO. Descriptive statistics were used, but no statistical software were used to analyse data. Presentation of data was unclear, and the coherence between data and results was poor. | 80% HIGH QUALITY |
| Okonofua | 2005 | Okonofua FE, Shittu SO, Oronsaye F, Ogunsakin D, Ogbomwan S, Zayyan M. Attitudes and practices of private medical providers towards family planning and abortion services in Nigeria. Acta Obstetricia et Gynecologica Scandinavica. [Online] 2005;84(3): 270–280. Available from: doi:10.1111/j.0001-6349.2005.00405.x | YES. Non-probability: All private medical practitioners were chosen for study | YES. All providers in the population were contacted for survey, reasons were given for providers excluded from study | YES. Questionaire was pretested and validated. Variables had clear definition. | YES. Estimated 8% non-response. | YES. SPSS package was used for data analysis. Regression analysis was used to understand variables influencing practice. | 100% HIGH QUALITY |
| Tran | 2010 | Tran NT, Jang MC, Choe YS, Ko WS, Pyo HS, Kim OS. Feasibility, efficacy, safety, and acceptability of mifepristone-misoprostol for medical abortion in the Democratic People's Republic of Korea. International Journal of Gynecology & Obstetrics. [Online] 2010;109(3): 209–212. Available from: doi:10.1016/j.ijgo.2010.01.012 | YES. Non-probability: Patients who visited the facility at a certain time were chosen for the study. | YES. Clear inclusion and exclusion criteria to choose patients, and explanation was given as to why eligible women were not included in study. Efforts were made to collect missing data. | NO. Variables had clear definition, but measurements were not justified by authors, and justify use of questionnaires. | YES. No women were lost to follow-up in the study. | YES. Data were analysed using Epi Info, chi-sq or figher exact test were used to analyse categorical data and t-test was used for continuous variables. | 80% HIGH QUALITY |

### **Mixed Method Studies**

|  |  |  | **1. QUALITATIVE STUDIES** | | | | |
| --- | --- | --- | --- | --- | --- | --- | --- |
| Author | Year | Reference | 1.1. Is the qualitative approach appropriate to answer the research question? | 1.2. Are the qualitative data collection methods adequate to address the research question? | 1.3. Are the findings adequately derived from the data? | 1.4. Is the interpretation of results sufficiently substantiated by data? | 1.5. Is there coherence between qualitative data sources, collection, analysis and interpretation? |
| Banerjee et al. | 2010 | Banerjee SK, Andersen K, Tank J, Parihar M, Shah M, Thanwala U. Evaluation of a network of medical abortion providers in two districts of Maharashtra, India. Global Public Health. [Online] 2011;6(3): 283–292. Available from: doi:10.1080/17441692.2010.516758 | CAN'T TELL. Authors barely explained the rationale for their qualitative approach in this study | YES. Data collection via interview in the form of tape recording while conducting a questionaire with interviewer | YES. Qualitative responses were coded manually and merged into the data-set. Only one quote from an interviewee was included. | YES. Quote included in this study could justify the interpretation of results. | Yes. There was coherence between qualitative data collection, analysis and interpretation. |
| Kawonga | 2008 | Kawonga M, Blanchard K, Cooper D, Cullingworth L, Dickson K, Harrison T, et al. Integrating medical abortion into safe abortion services: experience from three pilot sites in South Africa. Journal of Family Planning and Reproductive Health Care. [Online] 2008;34(3): 159–164. Available from: doi:10.1783/147118908784734846 | YES. Rationale was clearly stated as to why qualitative method was chosen. | CAN'T TELL. Study did not explain how qualitative data were analysed. | CAN'T TELL. Study did not explain findings were inferred from qualitative data. | NO. Most interpretations were not justified with qualitative data. | YES. There was coherence between qualitative collection, analysis and interpretation. |

|  |  |  | **3. NON-RANDOMIZED STUDIES** | | | | |
| --- | --- | --- | --- | --- | --- | --- | --- |
| Author | Year | Reference | 3.1. Are the participants representative of the target population? | 3.2. Are measurements appropriate regarding both the outcome and intervention (or exposure)? | 3.3. Are there complete outcome data? | 3.4. Are the confounders accounted for in the design and analysis? | 3.5. During the study period, is the intervention administered (or exposure occurred) as intended? |
| Banerjee et al. | 2010 | Banerjee SK, Andersen K, Tank J, Parihar M, Shah M, Thanwala U. Evaluation of a network of medical abortion providers in two districts of Maharashtra, India. Global Public Health. [Online] 2011;6(3): 283–292. Available from: doi:10.1080/17441692.2010.516758 | CAN'T TELL. It is unclear if the 87 doctors and the 514 patients who visited these doctors in this study were representative of the study population. There was a clear description of doctors included in study, but no information on patients. Although authors did not state exclusion critera, they explained clearly why the doctors were chosen to receive intervention and gave reasons for non-participation of doctors. | YES. Variables were clearly defined and accurately measured. The measurements appropriate for answering research question, although some explanation for justification of measurements would be desirable. The measurements reflect what they are support to measure; measures of intervention and outcome were pre-tested and deemed valid | YES. Almost all participants contributed to the all the measures: 88% response at post-intervention survey | NO: Provider responses were not matched before and after intervention. In addition, there was no control group for comparison | YES. However, study did not state if there were presence of unplanned co-interventions that may influence outcome. |

|  |  |  |  |  |  |  |  |
| --- | --- | --- | --- | --- | --- | --- | --- |
|  |  |  | **4. QUANTITATIVE DESCRIPTIVE STUDIES** | | | | |
| Author | Year | Reference | 4.1. Is the sampling strategy relevant to address the research question? | 4.2. Is the sample representative of the target population? | 4.3. Are the measurements appropriate? | 4.4. Is the risk of nonresponse bias low? | 4.5. Is the statistical analysis appropriate to answer the research question? |
| Kawonga | 2008 | Kawonga M, Blanchard K, Cooper D, Cullingworth L, Dickson K, Harrison T, et al. Integrating medical abortion into safe abortion services: experience from three pilot sites in South Africa. Journal of Family Planning and Reproductive Health Care. [Online] 2008;34(3): 159–164. Available from: doi:10.1783/147118908784734846 | YES. Non-probability: Patients who visited the facility at a certain time were chosen for the study. | YES. Clear inclusion and exclusion were stated in study, and explanation was given as to why eligible individuals were not included in study. Efforts were made to collect missing data. | YES. Standardised instruments were used for data collection. Measurements are justified and appropriate for answering research question. | YES. 10% patients were follow-up in the study. | YES. SPSS Statistical package was used to analyse data. Measures of spread was used where necessary. |

|  |  |  | **5. MIXED METHODS STUDIES** | | | | |
| --- | --- | --- | --- | --- | --- | --- | --- |
| Author | Year | Reference | 5.1. Is there an adequate rationale for using a mixed methods design to address the research question? | 5.2. Are the different components of the study effectively integrated to answer the research question? | 5.3. Are the outputs of the integration of qualitative and quantitative components adequately interpreted? | 5.4. Are divergences and inconsistencies between quantitative and qualitative results adequately addressed? | 5.5. Do the different components of the study adhere to the quality criteria of each tradition of the methods involved? |
| Banerjee et al. | 2010 | Banerjee SK, Andersen K, Tank J, Parihar M, Shah M, Thanwala U. Evaluation of a network of medical abortion providers in two districts of Maharashtra, India. Global Public Health. [Online] 2011;6(3): 283–292. Available from: doi:10.1080/17441692.2010.516758 | CAN'T TELL. Authors did not state rationale for using mixed-methods | YES. Data collection of both qualitative and quantitative information was well integrated. | NO. There was little integration of qualitative results in the study, qualitative data seems to have little value add to study. | CAN'T TELL. Methods did not mention anything on divergences or how they were addressed. | YES. Overall grade. |
| Kawonga | 2008 | Kawonga M, Blanchard K, Cooper D, Cullingworth L, Dickson K, Harrison T, et al. Integrating medical abortion into safe abortion services: experience from three pilot sites in South Africa. Journal of Family Planning and Reproductive Health Care. [Online] 2008;34(3): 159–164. Available from: doi:10.1783/147118908784734846 | YES. The authors stated the rationale for using both methods. | YES. Qualitative data formed the picture from provider's perspective, while quantitative data formed the picture from patient's perspective | YES. Interpretation of both type of data is well integrated | YES. There were no divergence. | NO. Qualitative did not meet criteria. |

1. WHO recommendation 400mg mifepristone, followed by 800mg misoprostol 24 hours later [22]. [↑](#footnote-ref-1)
2. Quality was judged based on MMAT Critical Appraisal Tool – study methods were evaluating against the MMAT checklist, consisting 5 items (Appendix S3). High quality papers met at least 4 criteria; medium quality papers met 2 or 3 criteria; low quality papers met 1 criterion only. Refer to appendix for detailed evaluation of each criteria. [↑](#footnote-ref-2)
